# Thermal physiology integrated species distribution model predicts profound habitat fragmentation for estuarine fish with ocean warming

**DOI:** 10.1038/s41598-022-25419-4

**Published:** 2022-12-16

**Authors:** Akila Harishchandra, Huijie Xue, Santiago Salinas, Nishad Jayasundara

**Affiliations:** 1 School of Marine Sciences, The University of Maine, 168 College Ave, Orono, ME 04469 USA; 2 State Key Laboratory of Marine Environmental Science, College of Ocean and Earth Sciences, Xiamen University, Xiang’an South Road, Xiamen, 361102 China; 3 327 Dow Sciences Center, Kalamazoo College, 1200 Academy Street, Kalamazoo, MI 49006 USA; 4 Nicholas School of the Environment, Duke University, 9 Circuit Drive, Durham, NC 27710 USA

**Keywords:** Climate sciences, Ecology, Ocean sciences

## Abstract

Species distribution models predict a poleward migration for marine ectotherms with ocean warming. However, a key limitation in current species distribution models (SDM) is that they do not account for population-specific heterogeneity in physiological responses to temperature change resulting from local adaptations and acclimatization. To address this gap, we developed a novel, Physiology Integrated BioClimate Model (PIBCM) that combines habitat-specific metabolic thermal physiological tolerance of a species into a bioclimate envelope model. Using a downscaling approach, we also established a fine-resolution coastal sea-surface temperature data set for 2050–2080, that showed a high degree of location-specific variability in future thermal regimes. Combining predicted temperature data with the PIBCM model, we estimated habitat distribution for a highly eurythermal intertidal minnow, the Atlantic killifish (*Fundulus heteroclitus*), a species that likely presents a best-case-scenario for coastal vertebrates. We show that the killifish northern boundary shifts southwards, while distinct habitat fragmentation occurs in the southern sub-population (due to migration of adjacent fish populations to the nearest metabolically optimal thermal habitat). When compared to current SDMs (e.g., AquaMaps), our results emphasize the need for thermal physiology integrated range shift models and indicate that habitat fragmentation for coastal fishes may reshape nursery habitats for many commercially and ecologically important species.

## Introduction

Rising global ocean temperature will profoundly shift habitat distribution patterns for aquatic ectotherms. This has generated significant interest in predictive species distribution models (SDMs)^1–3^ to infer habitat suitability and range by combining species’ spatial extent with environmental data^4,5^. Climate envelope modeling^6^ is a SDM technique that uses a species' present geographical distribution to determine its environmental niche and to predict the re-distribution patterns based on future climatic conditions^7^.

There are two key limitations in correlative species distribution models. First, most models consider the minimum and maximum habitat temperatures of the entire population range as the upper and lower thermal limits of a given population, without accounting for the local adaptation of sub-populations^8,9^. Secondly, existing models do not consider species’ capacity to modify their thermal physiological properties, i.e., acclimatization^10–13^. Hybrid correlative models have addressed some of these limitations^14–16^ by integrating thermally dependent phenotypic parameters (e.g., development threshold for larvae^16^, growth rates, and clearance rate^14^) into model predictions). These approaches still do not consider within-species physiological variation, (e.g., local thermal profile dependent organismal physiological acclimation capacity) at a high spatial resolution.

Here, we postulate that using fundamental cellular physiological processes (e.g., energy metabolic rates) can yield more accurate predictions of a population’s response to changing thermal habitats than whole-organism parameters like growth rate. Aerobic scope—the absolute difference between the maximum metabolic rate (MMR) and routine metabolic rate (RMR)—is a highly thermally-dependent parameter, that governs a species’ reproductive success, growth, survival, and biogeography^11,17–22^. This notion is captured in the oxygen and capacity-limited thermal tolerance (OCLTT) framework^19,20,23,24^, which indicates that declining aerobic performance measured in terms of aerobic scope (AS) results in a thermal limitation of ectotherms at both ends of the thermal envelope. Despite criticism of the overall framework^25^, the highest level of aerobic scope is considered to be tightly linked to optimum performance^26^. Studies have integrated these concepts to predict the future metabolic changes in terrestrial and aquatic ectotherms with global warming^27,28^, and analyses on predicting species range shifts are lacking.

**Figure 1:**
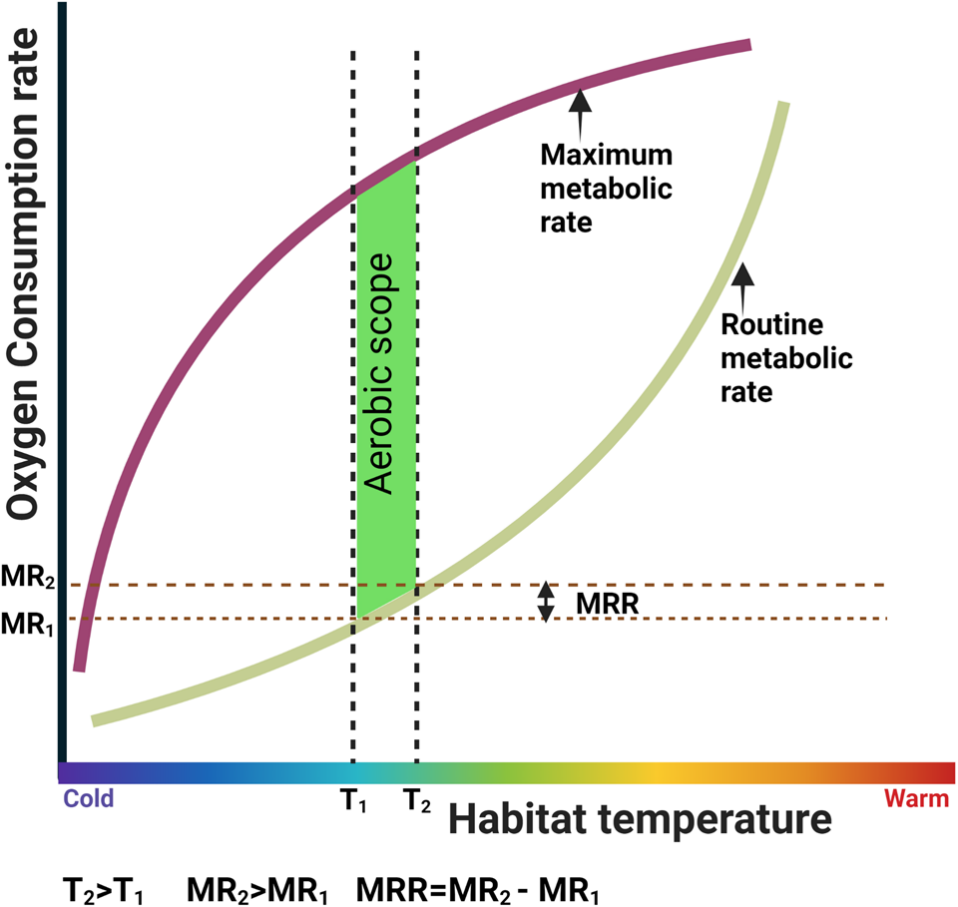
The physiological theory underpinning physiology integrated bioclimate envelope model. Conceptual depiction of metabolic rate range (MRR) used to integrate Atlantic killifish thermal tolerance into the physiology integrated bio-climate model. Theory predicts that the routine metabolic rate (blue line) of an ectothermic teleost exponentially increases with the increasing temperature while the maximum metabolic rate (brown line) plateaus with increasing temperature and that the ideal thermal window (T_1_ –T_2_) is where aerobic scope (difference between the maximum and routine metabolic rates) is maximized. We postulate that to maintain maximum aerobic scope following an increase in temperature, a fish from a given habitat will modify their thermal physiological properties to stay within a certain routine metabolic rate range (MRR—the difference in routine metabolic rates between T_1_ and T_2_) (Created with BioRender.com).

Here, we propose a novel framework to integrate the thermal plasticity of aerobic metabolism into a bioclimate envelope model and predict range shifts of marine ectotherms at a high spatial resolution. The central premise of our model is that a marine ectotherm maintains its routine metabolic rates within a certain habitat temperature range (at the climatological averaged minimum and maximum habitat temperature of the given location, i.e., T_1_ and T_2_ in Fig. 1) in its current thermal environment, where it can sustain the highest possible aerobic scope at that location. Changes in the current thermal environment could reduce the optimum aerobic scope (explained in the OCLTT theory), leading to a habitat range shift unless the organism acclimates to the rising habitat temperatures. In this study, we hypothesized that the routine metabolic rates range (the range between routine metabolic rates calculated at the minimum and maximum habitat temperatures; defined as Metabolic Rate Range—MRR, Fig. 1) is a representative parameter of the optimum aerobic scope in a given location for a marine ectotherm. In other words MRR is a proxy for aerobic scope range in a given location. We determined MRR in a given location by calculating the absolute difference between routine metabolic rates at the climatological maximum and minimum habitat temperatures in that location over the last 37 years (Fig. 1). For this, we use the Metabolic Theory of Ecology equation (MTE)^29,30^, which explains the thermal dependence on aerobic metabolism accounting for body size and environmental temperature (Eq. 1).

1 $$\mathrm{B}={\mathrm{b}}_{\mathrm{o}}{\mathrm{M}}^{3/4}{\mathrm{e }}^{\frac{-\mathrm{E}}{\mathrm{KT}}}$$

Here, B is resting/routine metabolic rate, ${b}_{o}$ is an empirically derived taxon-specific normalization constant (8.617 × 10^–5^ eV/K), M is body mass, E is the averaged enzyme activation energy in eV, K is Boltzmann constant, and T is the habitat temperature in Kelvin. Importantly, the averaged enzyme activation energy (E) is the thermally dependent physiological variable that can be modified through acclimation, developmental, transgenerational plasticity, and/or local adaptation. Although it was initially assumed a universal temperature dependence for E, indicating that it is constant within a taxonomic group (e.g., 0.43 for fish)^29^, ectotherms may modulate their E value^31,32^. Accordingly, here we posit that under thermal stress ectotherms may modify E to maintain a constant MRR in the current habitat.

**Figure 2:**
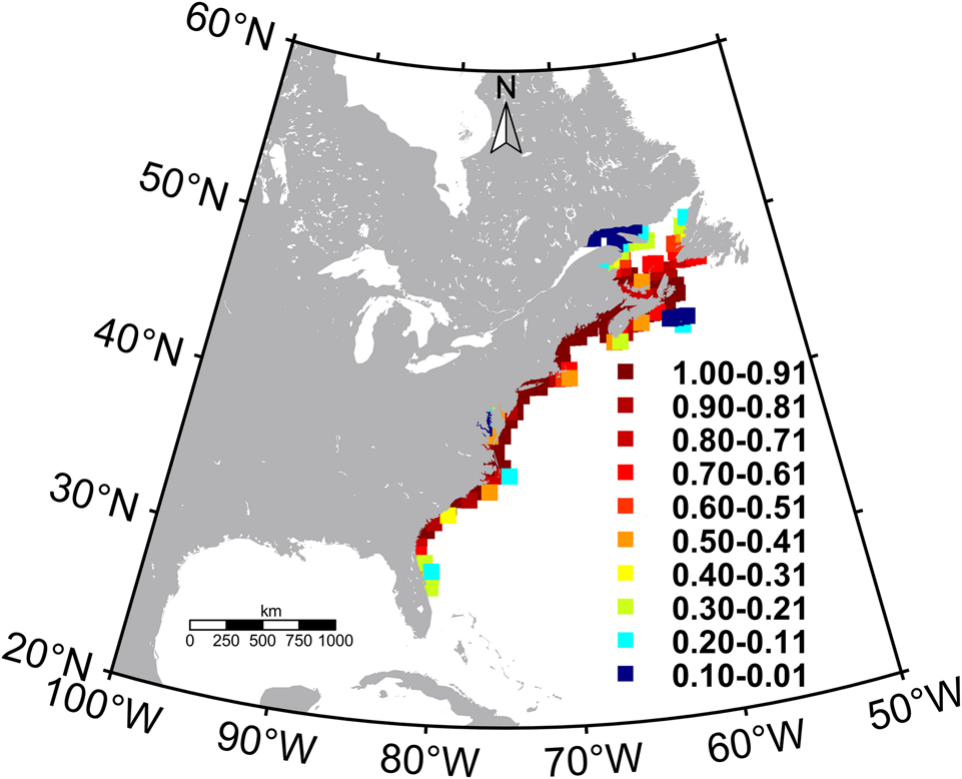
Atlantic killifish probability habitat distribution within the native range. (Source: AquaMaps). The figure was created using m_map toolbox v1.4 (https://www.eoas.ubc.ca/~rich/map.html) in MATLAB version R2022a (University of Maine academic license 358411) (https://www.mathworks.com/).

To develop our physiology-integrated bio-climate model (PIBCM), we first tested the species specificity of the E value across a range of fish species. We show that this value is highly species-dependent and sensitive to the population’s local thermal envelope. We then simulated metabolic rate sensitivity to E in the MTE equation to demonstrate that metabolic rates and the metabolic rate ranges shift with E value changes. Then we quantified the habitat range shift between 2040–2069 and 2070–2099 for a highly eurythermal intertidal minnow, the Atlantic killifish (*Fundulus heteroclitus*). We calculated the current MRR using climatological monthly minimum and maximum sea surface temperature (SST) data over the last 37 years for killifish in their current local habitat at a resolution of 0.5°. We parameterized the model for the two subspecies of Atlantic killifish that live along the North American East Coast^33,34^ (Fig. 2). The southern population is more heat tolerant (7–31 °C range) than the northern population (− 1.4 to 21 °C range)^33^, and the daily and seasonal temperature fluctuations vary widely between populations^33,35^. Thus, we calculated E values for a range of acclimation temperatures for northern and southern killifish based on data from an extensive killifish acclimation experiment^36^. To calculate MRR values associated with future projects, we substituted contemporary SSTs with predicted SSTs based on a novel coastal SST dataset we developed for the 2050s and 2100s. We implemented a downscaling approach built on averaging three coupled model intercomparison project phase 5 (CMIP5) model outputs (Representative Concentration Pathways (RCP)2.6,4.5,8.5; Supplementary Table S1). Subsequently, the model allowed a fish population in a given habitat (0.5° grid cells) to remain in the same grid if the fish can modulate the E value at the predicted future ocean temperature. If not, the model will find the nearest grid location with the same or lesser MRR as the best habitat for that given fish population to relocate with the ocean warming.

To our knowledge, this is the first study to integrate thermal physiological acclimation into SDMs, and our use of an eurythermal species like the killifish could represent a best-case scenario for other coastal and estuarine fishes in response to future warming.

## Results

### Species-specific biochemical activation energies—E values

**Figure 3:**
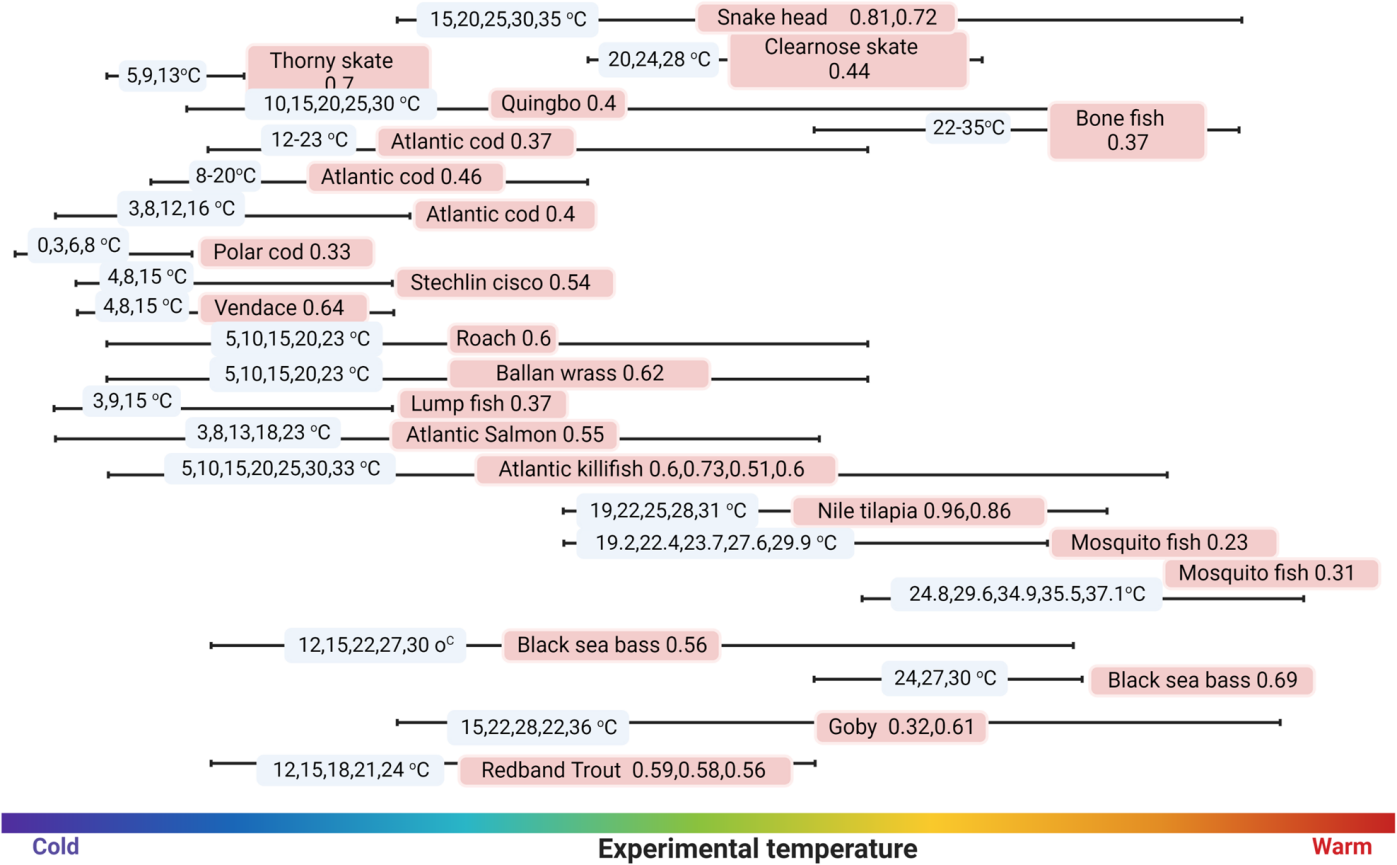
Summary of the species-specific E values calculated for 19 fish species. Same fish species may have different E values depending on the experimental conditions and temperature ranges. The solid lines depict the experimental temperature range, and the blue legends show the temperature values. Fish species name and the estimated E values (in eV) are displayed in the pale red boxes (Created with BioRender.com).

Species-specific E values for 19 teleost fishes (five freshwater, seven marine, four brackish, and three euryhaline fish) were calculated using published standard or routine metabolic rates (SMR/RMR) (Table 1 and Fig. 3). E values were highly species-specific and ranged from 0.23 (mosquitofish, *Gambusia affinis*) to 0.96 eV (Nile tilapia, *Oreochromis niloticus*), and depends on acclimation temperature (Table 1 and Fig. 3). The mean E value calculated in our study (0.55 eV $\pm$ 0.17) was lower than previous estimations for all taxa (0.62 eV) and higher than fish-specific E value (0.433 eV)^29^. Within-species differences in E value were clearly reflected in comparisons between northern and southern killifish subpopulations, confirming that acclimation and local adaptions significantly alter these values^36^, (Table 1). Acclimated and acutely exposed northern Atlantic killifish E values were lower than that of the Southern populations. Overall, these data demonstrate the species specificity of the E value and its dependence on the population’s thermal history.

**Table 1:** Summary of the species-specific E values calculated for 19 fish species.

	E value (eV)	R^2^	P	Experiment temperature (°C)	Mean Weight (g)	Environment	References
Redband Trout	0.59	0.57	< 0.001	12,15,18,21,24	1.14	F*	^65^
	0.58	0.53	< 0.001		2.41		
	0.56	0.49	< 0.001		3.4		
Goby	0.32	0.28	< 0.001	15,22,28,33,36	1.15	M^¶^, B^ℾ^	^66^
	0.61	0.3	< 0.001		0.73		
Black sea bass	0.69	0.74	< 0.001	24,27,30	265.6	M	^67^
	0.56	0.87	< 0.001	12,17,22,27,30	375		
Mosquito fish	0.31	0.04	0.0052	24.8,29.6,34.9,35.5,37.1	50.57	F, B	^68^
	0.23	0.02	0.062	19.2,22.4,23.7,27.6,29.9	70.38		
Nile Tilapia	0.96	0.95	< 0.001	19,22,25,28,31	50	F, B	^69^
	0.86		< 0.001		200		
Atlantic Killifish	0.6	0.85	< 0.001	5,10,15,20,25,30,33	4.58	M, B, F	^36^
	0.73	0.81	< 0.001		6.14		
	0.51	0.9	< 0.001		6.26		
	0.6	0.91	< 0.001		6.77		
Atlantic Salmon	0.55	0.9	< 0.001	3,8,13,18,23	443.77	M, B, F	^70^
Lump fish	0.37	0.54	< 0.001	3,9,15	310.21	M	
Ballan Wrass	0.62	0.14	< 0.001	5,10,15,20,23	162.59	F	^71^
Roach	0.6	0.24	< 0.001	5,10,15,20,23	63.76	B, F	
Vendace	0.64	0.4	< 0.001	4,8,15	30.78	M, B, F	
Stechlin cisco	0.54	0.44	< 0.001	4,8,15	16.15	F	
Polar cod	0.33	0.42	< 0.001	0,3,6,8	16	M	^72^
Atlantic cod	0.4	0.68	< 0.001	3,8,12,16	40.76	M	
Atlantic cod	0.46	0.63	< 0.001	8,20	73	M	^73^
	0.37	0.51	< 0.001	12,23	64.6		
Bone fish	0.37	0.1	0.013	22,35		M	^74^
Quingbo	0.4	0.71	< 0.001	10,15,20,25,30	2.87	F	^75^
Thorny Skate	0.7	0.33	0.0018	5,9,13	1.56	M	^76^
Clearnose Skate	0.44	0.26	0.01	20,24,28	1.22	M	
Snakehead	0.81	0.95	< 0.001	15,20,25,30,35	4.4	F	^77^
	0.72	0.91	< 0.001		4.42		

Fish from the same species may have different E values depending on the experimental conditions used to estimate the fish’s routine metabolic rates. Least square regression was applied to regress the natural log-transformed fish routine or standard metabolic rates with the inverse temperature.

*F-Fresh water.

^ℾ^B-Brackish water.

^¶^M-Marine.

### Sensitivity analysis

To determine the effects of different E values on the metabolic rate and metabolic rate range of a given organism, we examined the relationship between the E value and metabolic rate based on a sensitivity analysis of the MTE equation. Hypothetical metabolic rates were calculated as a function of temperature (0–40 °C) and body mass (1–1000 g) for E values between 0.01 and 1 eV. Metabolic rate can range from − 10.95 to 9.6 (log10 mW) (Supplementary Fig. S1A). As expected from the MTE equation, a small variation (e.g., 0.1 eV) in the E value resulted in a 70–40-fold change in metabolic rate across the temperature range used for metabolic rate calculations (0–40 °C) (Supplementary Fig. S1A). Metabolic rates generally increased with increasing temperature and body size as expected, but the changes in the E value had the greatest impact on metabolic rates (Supplementary Fig. S1B,C). The effect of size (weight) on metabolic rate was highest at very small size ranges (~ 1 g–100 g), and this effect decreased as the animals got larger (Supplementary Fig. S1B). As such, the impact of a 0.1 eV change in the E value on metabolic rate is equivalent to a 70-fold change in size for a given organism. For a 10 g fish, the effect of temperature on metabolic rate was minimal at higher E values but increased with increasing E values, i.e., the slope of the line between metabolic rate and body mass decreased with decreasing E values (Supplementary Fig. S1C). Calculated metabolic rate ranges (MRR) between two consecutive temperatures (e.g., 0–2 °C, 2–4 °C, 4–6 °C) increased linearly (Supplementary Fig. S1D), except for the MRR calculated at the lowest E value (0.01 eV).

Overall, these analyses confirm that metabolic rate is highly sensitive to the E value and metabolic rates of organisms maintaining higher E values are most sensitive to their habitat temperature. Killifish E values range between 0.5 and 0.7 eV (Table 1), suggesting moderate thermal sensitivity of metabolic rate, especially in the Northern populations. Furthermore, at this range of E values, MRR increases with increasing temperature (e.g., MRR is higher for a fish at 20–22 °C compared to a fish at 18–20 °C). This supports the notion that killifish may adjust their E value to return to their optimum MRR if habitat temperatures were to increase.

### Contemporary coastal SST variability in the study area

**Figure 4:**
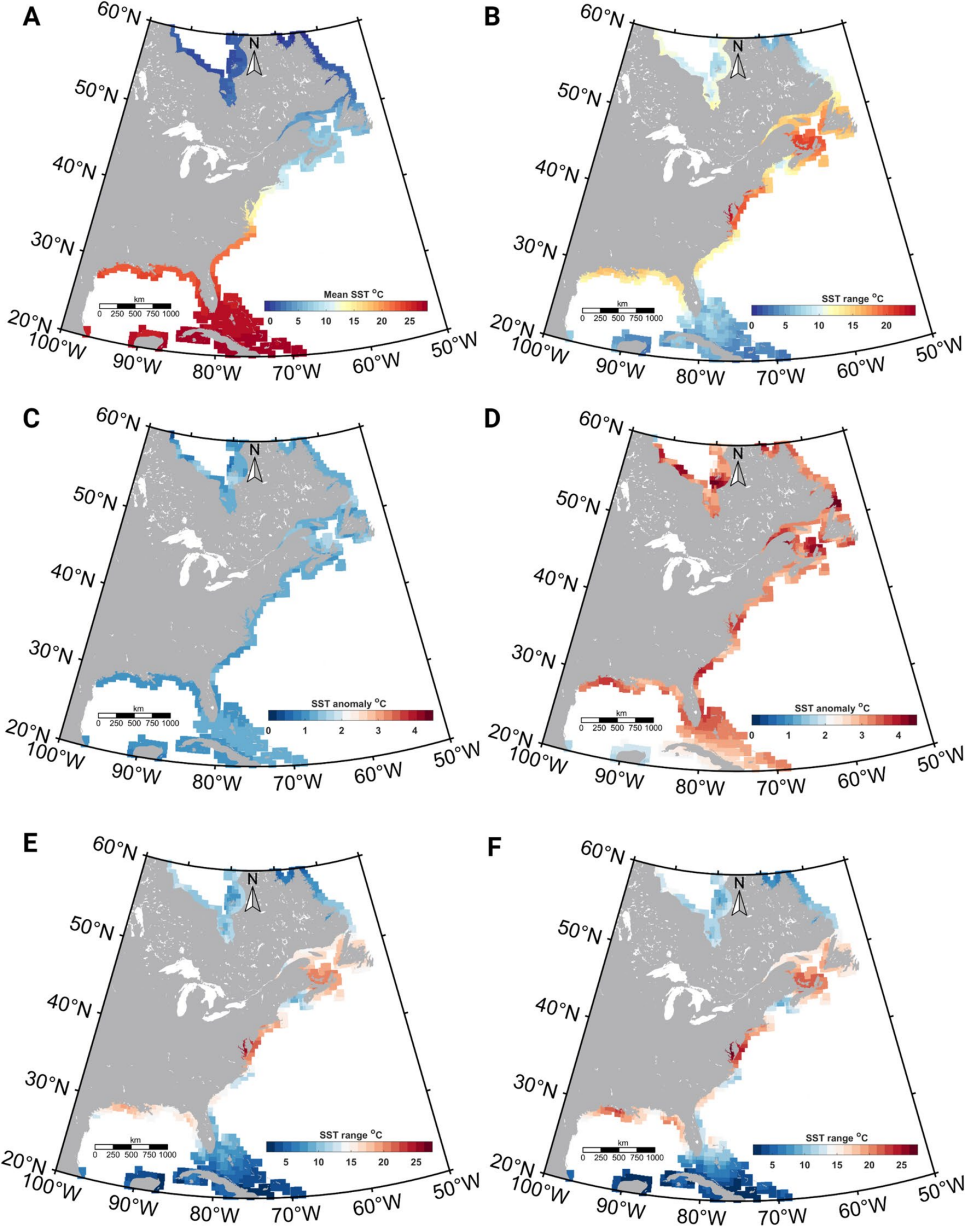
The contemporary and the climate model projected future sea surface temperature (SST) variability along the East Coast of North America (**A**) Climatological mean SST distribution along the North American East coastline during the contemporary (1982–2018) period (Copernicus data). (**B**) The SST range (Difference between maximum and minimum SST) along the same region and period. (**C**) SST anomaly (difference between the future and contemporary SST distribution) in the 2050s (RCP 2.6) (**D**) SST anomaly in the 2080s (RCP 8.5) (**E**) The SST range along the North American coastline during the 2050s (RCP 2.6) and (**F**) 2080s (RCP 8.5). Visualizations of all RCP scenarios are in the supplemental section (Supplementary Figs. 2 and 3). Figures were created using m_map toolbox v1.4 (https://www.eoas.ubc.ca/~rich/map.html) in MATLAB version R2022a (University of Maine academic license 358411) (https://www.mathworks.com/).

As expected, climatological mean (1982–2018) SST along the North American coastline showed a distinct latitudinal gradient (Fig. 4A). The highest and lowest mean SSTs (28.19 and − 1 °C) were recorded for 20.25 N (Florida Keys) and 59.75 N (Newfoundland and Labrador coast) respectively. Within the native Atlantic killifish habitat distribution (28–52 N), climatological mean SST extended from 1.54 to 24.29 °C. Location-specific climatological mean SST range (the difference between the maximum and minimum mean SST for a given location) spanned between 8 and 24.3 °C in the current killifish habitats (between 28 and 52 N), indicating a potential preference for thermally variable environments (Fig. 4B). The highest SST range (24.29 °C) was recorded in the Chesapeake Bay, while the SST range experienced by killifish was generally higher for the populations in the Delaware Bay region, and the southern Gulf of St. Lawrence coastal region.

### The climate model projected coastal SST variability

Our novel downscale approach to obtaining predicted SST showed a distinct latitudinal thermal gradient similar to the contemporary pattern and predicted a positive mean SST anomaly between contemporary and future periods (the 2050s and 2080s) under all RCP scenarios (Fig. 4C,D) and Supplementary Fig. S2). The location-specific SST range (the difference between the maximum and minimum climatological mean SST for a given location) in the 2050s and 2080s also showed a similar pattern to its contemporary distribution (Fig. 4E,F, and Supplementary Fig. S3). For the 2050s (RCP 2.6) and 2080s (RCP 8.5), the predicted highest SST range was 25.52 °C and 27.34 °C respectively, and was recorded in the Chesapeake Bay region (38.75 N,76.25 W) (Fig. 4E,F). Overall, our downscaled model output predicted an increasing SST range.

### Atlantic killifish thermal envelope-specific E values

**Figure 5:**
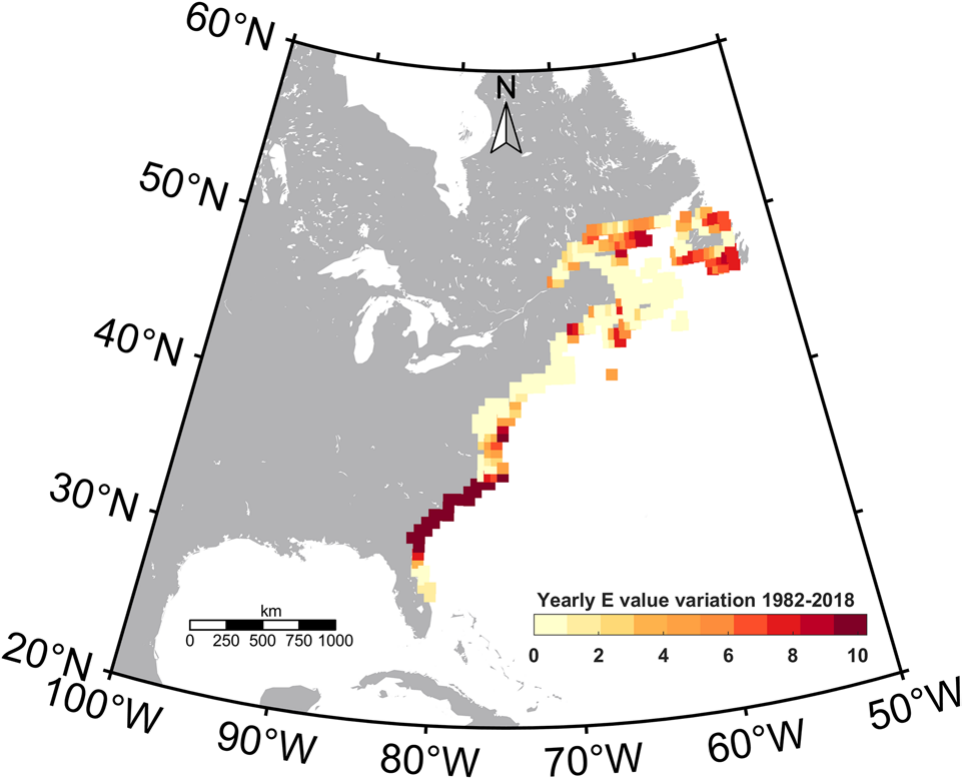
Yearly Atlantic killifish E value variation (10^3^) during the 1982–2018 period. Respective E values (eV) for each year’s maximum and minimum SSTs along the Atlantic killifish native habitat range were calculated using Healy and Schulte,2012 data. The figure was created using m_map toolbox v1.4 (https://www.eoas.ubc.ca/~rich/map.html) in MATLAB version R2022a (University of Maine academic license 358411) (https://www.mathworks.com/).

Based on location-specific SST ranges, we determined thermal habitat envelopes for killifish. The killifish metabolic rates we adopted for our study were estimated at a temperature array within 5 °C intervals (5,10,15,20,25,30 and 33 °C^36^. Therefore, we rounded up the long-term-averaged minimum and maximum habitat temperatures to the nearest 5 °C (Supplementary Table S2) to define all the possible killifish thermal envelopes. Essentially a given thermal envelope reflects the maximum and minimum temperatures of a given location along the current killifish habitat. We estimated nine thermal envelopes for the Northern subpopulations’ habitat range, and six thermal envelopes for the Southern subpopulation (Supplementary Table S3) and found three thermal envelopes (5–25 °C, 5–30 °C, and 10–30 °C) to be common for both populations. Six thermal envelopes (5–10 °C, 5–15 °C, 5–20 °C, 10–15 °C, 10–20 °C, and 10–25 °C) were unique to the Northern population range, while 15–30 °C, 20–30 °C, and 25–30 °C were unique to the Southern population range. E values were estimated for each of the thermal envelopes, and we found E values for the common thermal envelopes were higher in the Southern subpopulation than in the Northern subpopulation (Supplementary Table S3), further confirming that Southern subspecies are more thermally sensitive than the Northern subspecies. We then used the same approach to calculate thermal envelopes and their respective E values based on the predicted future temperatures. Overall, the metabolic rates, and respective metabolic rate ranges in each grid varied as a function of the thermal envelope and the envelope-specific E values. The yearly E value variance across the Atlantic killifish native range during the contemporary period showed a higher variance at the northern and southern ends (Fig. 5) and an evenly lower variance in the middle part. Notably, the southern habitat range showed the highest yearly E variance (Fig. 5) and the habitat fragmentation during the 2080s as predicted in our model.

### Predicted contemporary and future metabolic rate range (MRR) distribution

MRR, which is determined as a function of the population-specific E-value and contemporary or predicted future temperature change, is the deterministic physiological parameter in the climate envelope model. MRR within the Atlantic Killifish’s contemporary habitats ranged from − 7 to − 2 log10 mW. The highest killifish MRR was observed in the Northern limit of the contemporary habitat boundary around Nova Scotia and the surrounding coast (44 to 52 N) (Supplementary Fig. S4). In addition, patches of high MRR (~ − 2 log10 mW) were also observed within the contemporary habitat range (Supplementary Fig. S4). Overall, our downscaled SST data predicts three distinct clusters of MRR that can be categorized into low (− 8.5 to − 7 log10 mW), moderate (− 6 to − 5 log10 mW) and high (− 4 to − 3 log10 mW) MRR regions (Supplementary Fig. S5). With increasing temperatures in the 2050s (RCP 2.6) scenario, we observe a clear shift in these clusters, indicating an expansion of high MRR regions. This is particularly prominent in Nova Scotia, where the high MRR region expands its spatial extent. The coastal zone between 52 and 60 N is predicted to become a region with minimum MRR (~ − 10 to − 7 log10 mW) under future RCP scenarios.

### PIBCM predicted Atlantic killifish habitat shifts

**Figure 6:**
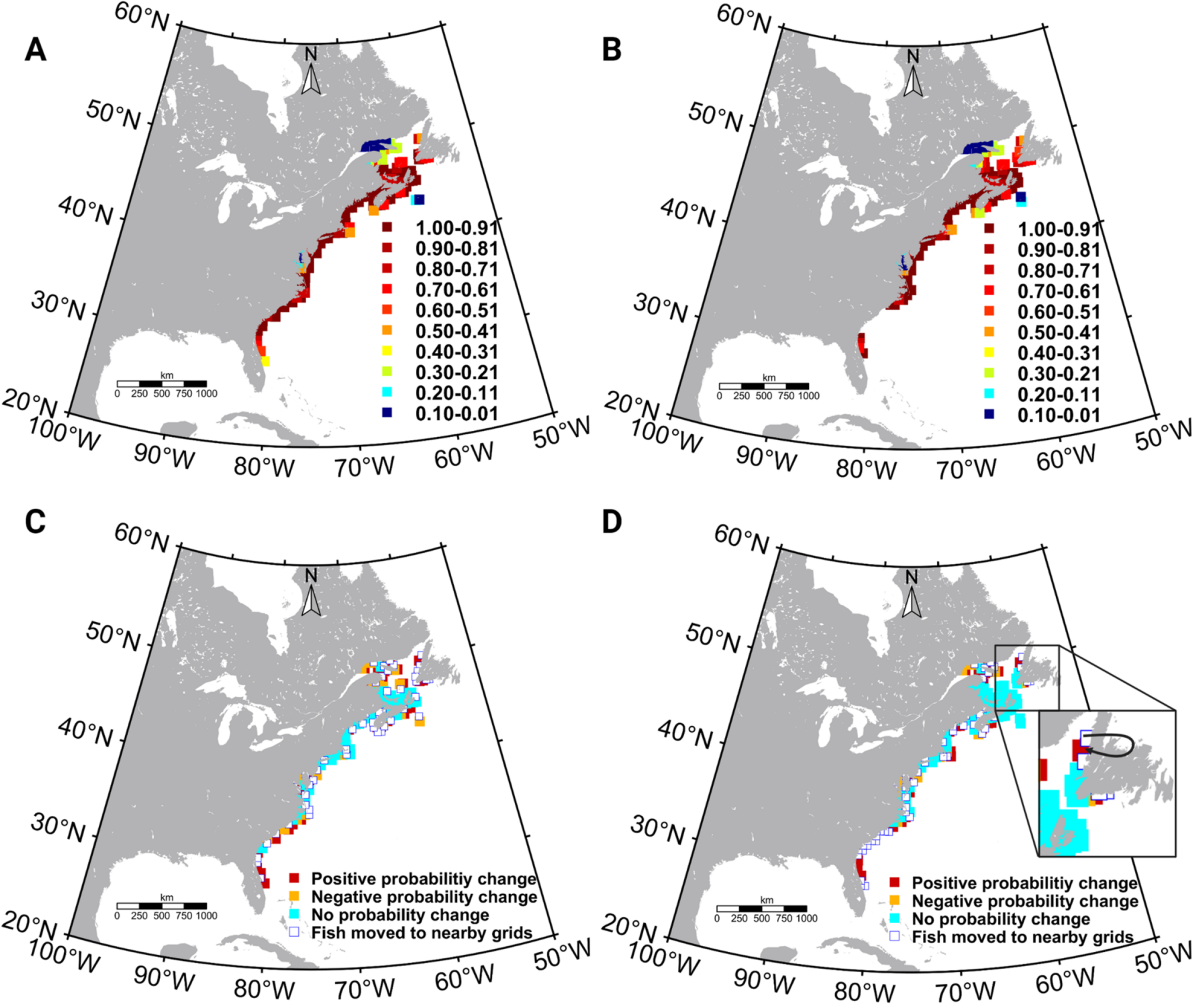
Physiology integrated model predicted killifish future distributions for two climate projections (representative concentration pathway (RCP)) and the comparison with contemporary distribution. **(A)** Atlantic killifish distribution (probability) in the 2050s (RCP2.6) and (**B**) 2080s (RCP8.5). (**C**) The comparison between new model predicted Atlantic killifish distribution for the 2050s (RCP2.6) and **(D)** the 2080s (RCP8.5) with the native habitat range. The dark arrows in the figure D shows the southward migration of the killifish populations inhabiting the northern most grid with ocean warming. Habitat predictions and comparisons for other RCP scenarios and time are displayed in the supplementary section (Supplementary Fig. S7 and S8). Figures were created using m_map toolbox v1.4 (https://www.eoas.ubc.ca/~rich/map.html) in MATLAB version R2022a (University of Maine academic license 358411) (https://www.mathworks.com/).

Three model conditions were implemented to predict the Atlantic killifish future habitat ranges (see “Methods” section). The first criterion is that if the maximum habitat temperature is less than 32 °C (physiological break temperature used for killifish in this model) and if the future maximum temperature is higher than the contemporary maximum temperature but future MRR is lower than current MRR, the fish population will remain in the same grid. Accordingly, PIBCM predicts that fish populations in several regions, including Newfoundland, Nova Scotia, New Brunswick, Cape Cod, Cape Hatteras, and the northern Florida coast will continue to stay in their current grids (Fig. 6A,B). In the Nova Scotia region (just south of Newfoundland), the number of killifish populations that met this criterion increased with time and the severity of RCP scenarios (Fig. 6C,D). This outcome is a result of the potential capacity of killifish to modify MRR by regulating their E value, even though the future maximum temperatures are higher than the contemporary maximum temperatures. The second criterion is that if the maximum habitat temperature is less than 32 °C and the future maximum temperature or MRR is lower than their contemporary values, the fish population will remain in the same grid. However, no killifish grid location followed this condition. The third criterion was that if the future maximum temperature exceeds 32 °C or if the future maximum temperature and future MRR exceed contemporary values, then the killifish population will move to the nearest grid locations with equal or lower MRR relative to their current habitat. ~ 78% of the grid cells representing killifish habitats followed this model condition (Supplementary Fig. S6). Under RCP 8.5, 8% of habitat grid cells exceeded the break temperature of 32 °C while 1% did so under RCP 4.5 in the 2080s (Supplementary Fig. S6D–F)).

We observed a combination of outcomes such as population shifts to their adjacent grid locations, habitat range contractions, and habitat fragmentations (Fig. 6A,B and Supplementary Fig. S7). We did not detect a clear northward range shift, and in fact, the northernmost killifish populations may shift to nearby southern grid locations (Fig. 6C,D). Under the RCP 2.6 2050s scenario, small-scale habitat fragmentations were predicted along the coastline (mainly due to the exceeding future MRR than the contemporary values), with the most pronounced changes in the southern part of the Gulf of Maine and the Cape Cod coast. The size of fragmentations widened in the 2080s and under different RCP scenarios. In particular, profound habitat fragmentations were observed for the Southern killifish subpopulations (Fig. 6B). This is a result of predicted habitat temperatures exceeding 32 °C, where some Southernmost killifish populations may aggregate around ~ 28 N–30 N seeking thermal refugia (Supplementary Figs. S6A–D, and S7A–D).

**Figure 7:**
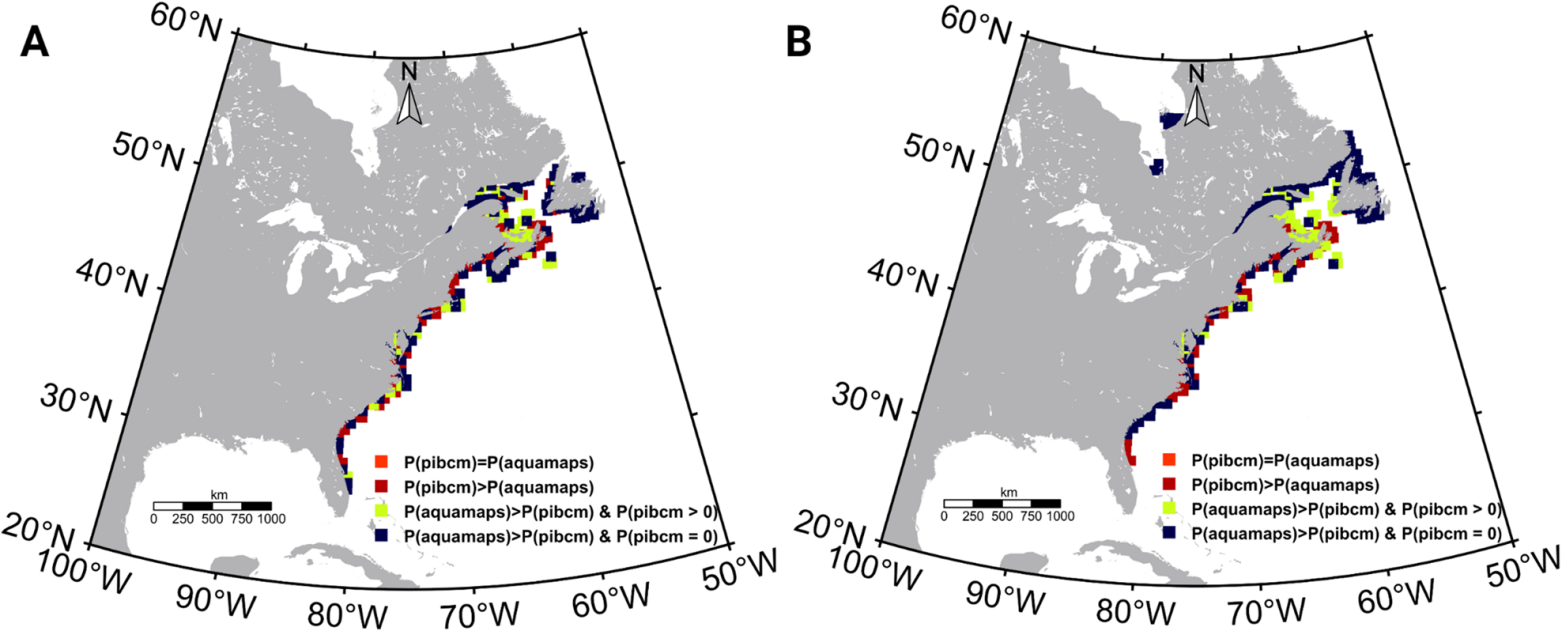
Comparison of Atlantic killifish habitat probabilities predicted in physiology integrated bio-climate model and AquaMaps model for two representative concentration pathway (RCP) scenarios. (**A**) Differences in the habitat probabilities predicted by the physiology integrated model and the traditional species distribution model (AquaMap) during the 2050s (RCP2.6) and 2080s (**B**) (RCP8.5). P(pibcm) is the predicted Atlantic killifish habitat probabilities by the physiology integrated model and P(aquamaps) means the predicted habitat probabilities by the AquaMaps model. Model comparisons for other RCP scenarios and times are displayed in the supplementary section (Supplementary Fig. S9). Figures were created using m_map toolbox v1.4 (https://www.eoas.ubc.ca/~rich/map.html) in MATLAB version R2022a (University of Maine academic license 358411) (https://www.mathworks.com/).

To quantify the negative and positive predicted aggregations to a given grid location, we calculated the difference in probability of a given grid cell being occupied by killifish under future scenarios compared to the contemporary distribution (Fig. 7 and Supplementary Fig. S8). Some grid cells that served as thermal refugia for nearby fish populations exceeded the cumulative probability when the contemporary grid probabilities shifted with the ocean warming and the model was set to adjust the exceeded probability value to 1. As such, Fig. 7 shows that in the 2050s (RCP 2.6) and 2080s (RCP 8.5), the probability of killifish inhabiting a given site remains unimpacted for some sites along the east coast, while some sites show reduced probability with habitat fragmentations.

### Comparison between AquaMaps and PIBCM predicted Atlantic killifish distribution

To compare the PIBCM predictions with a traditional bio-climate envelope model, we simulated an AquaMaps model for killifish distribution using climatological mean SST as the single environmental driver. According to AquaMaps predictions, the Northern and Southern boundaries of the Atlantic killifish native habitat will be expanded during the 2050s and 2080s (all RCP scenarios). The minimum northward range expansion was predicted for the 2050s (RCP2.6) and the maximum was in the 2080s (RCP8.5). The maximum range expansion was limited to the Canadian east coast at around 55° N. This contrasts with our PIBCM predicted habitat distributions, which show little northward expansion. When comparing the predicted population probability distribution by AquaMaps and PIBCM for a given RCP scenario (Supplementary Fig. S9), results showed contrasting likelihoods of killifish existence in a given habitat. Notably, as described earlier, the PIBCM model predicted habitat fragmentation, while AquaMaps did not (Supplementary Fig. S9A,B).

## Discussion

Our PIBCM indicates a complex habitat pattern shift that may lead to a series of disconnected killifish populations along the North American coastline in the future (Fig. 6C,D and Supplementary Fig. S6). Notably, in contrast to the killifish habitat poleward expansion predicted in the traditional bio-climate model (e.g., AquaMaps), the PIBCM predicted aggregations to thermally optimal sites including shifts by the Northernmost killifish populations to their nearest Southern grids (Fig. 6D). These predicted future habitat distribution patterns are likely to occur both over multiple generations as well as potentially within the lifetime of a fish.

Our model emphasizes the importance of integrating physiological responses into species distribution models. Most of the current habitat modeling techniques match organisms’ contemporary environmental niche with the future environmental conditions^1,2^ to predict future habitats but ignore organismal capacity to acclimatize. For example, a Generalized Additive Model that used SST as the primary parameter predicted an erroneous northward migration of *Calanus helgolandicus*^37^, inferring the likely importance of integrating physiological responses. To this end, our approach provides a novel framework for integrating organismal physiological responses into species distribution models. Here, metabolic rate range (MRR) was considered the fundamental predictive unit that decides the physiological affinity of a given Atlantic killifish population to its geographical range. The central premise of our model is that a marine ectotherm will maintain its routine metabolic rates within a certain range in its current thermal environment, to sustain the highest possible aerobic scope at that location. Instead of using aerobic scope directly, we used MRR for our calculations for several reasons. At the fundamental level of the model building, we considered a fish population in a given grid, maintains its optimum aerobic scope to inhabit the grid and the MRR (the difference between routine metabolic rates calculated at the maximum and minimum habitat temperatures) is a proxy of the maintained aerobic scope. Aerobic scope, the difference between the maximum and routine metabolic rates at a given temperature is considered a key physiological determinant of organismal fitness in a given habitat, and in this study, we postulated that the Atlantic killifish habitat probability in a given grid is linked to the grid-specific aerobic scope. However, the numerical calculation of aerobic scope is challenging across the temperature range we consider in our study, since there is no established mathematical equation to calculate maximum metabolic rates as a function of temperature. Therefore, we use the routine rate ranges calculated based on the MTE equation as a proxy for aerobic scope. This also enabled us to integrate the E value, which is indicative of the population-level biochemical capacity of ectotherms to acclimate to rising ocean temperatures. Additionally, although it is considered that organisms maintain their highest AS at the optimum temperature (T_opt_), the laboratory-measured T_opt_ to some extent may not be correlated with the organism’s preferred functional habit temperature^38^, and in several tropical fish species,’ optimum performance temperatures reflect more closely at the warmer end of their habitat temperature range^39^, that showed the ambiguities underlying the accurate estimation of the most appropriate temperature to determine the optimum habitat AS. Therefore, we anticipated that MRR provides a more robust physiological parameter to use as an indicator of optimum thermal range. Collectively, we posit here that, MRR serves as a useful deterministic physiological parameter, but further experiments are encouraged to prove its predictive capacity of species’ habitat distribution patterns.

We assumed that even with increasing SST, a fish population would remain in the same grid cell if future MRR is lower or equal to the current MRR if the fish can increase its E value through acclimation, developmental or transgenerational plasticity. Indeed, we show a wide range of E values for different teleost ectotherms, inferring the critical role of thermal acclimation capacity and local adaptations likely to play in determining thermal optimums^40,41^. Accordingly, the MRR calculations can serve as an informative physiological parameter that can be integrated into other bioclimate models as well as statistical species distribution modeling approaches such as generalized additive or linear models (GAM or GLM)^2,42^.

Our model relies on several key assumptions that warrant further experimental analyses. For example, the metabolic theory of ecology (MTE^29,30^) equation and the oxygen capacity-limited thermal tolerance theory (OCLTT^19,20,23,24^ are critical to our model. There are significant debates on OCLTT^25,43,44^ and MTE^31,32^ frameworks. One widely debated aspect of MTE is the scaling exponent. Here, we used 0.75 as the mass scaling factor^45^, although this value may depend on lifestyle, swimming mode, ambient temperature, and other environmental variables^46,47^. Previous studies have shown that the killifish scaling exponent is ~ 0.75^48^, validating our approach. While species-specific scaling exponents should be considered in PIBCM in future studies, bioenergetic outcomes of acclimation and local adaptation to abiotic environmental variables including temperature, are likely to be reflected via changes in the E value as also depicted by our sensitivity analysis (Supplementary Fig. S1). Despite the ambiguities around MTE and OCLTT frameworks, the two key attributes we focused on, aerobic scope (AS) optimization within a thermal window and the kinetics of the E value, are generally established. Here, we assumed that a given population optimizes AS by maintaining a routine metabolic rate within a certain range by regulating thermodynamic effects on enzyme activation energy (E). Importantly, we deviated from the MTE equation by postulating that the E value is species-specific and depends on the thermal history of a given population. For example, Kinnison et al*.*^49^ demonstrated fluctuations in E values of locally adapted chinook salmon resulting in a counter-gradient variation between these populations. This is further inferred in our analysis of data from published literature on 19 different fish species shows that the E value ranges between 0.23 eV and 0.96 eV. Moreover, our sensitivity analysis confirmed that a 0.1 eV change in the E value can have a significant effect on metabolic rate output. We posit that changes in the E value reflect biochemical factors sensitive to environmental temperature changes. Therefore, acquired thermal tolerance following thermal acclimation or developmental plasticity can alter the E value, providing a quantifiable mechanistic parameter for our model. However, there is limited information on precise physiological factors driving the E value^50^ and further research is warranted to examine a causal relationship between enzyme kinetic energy changes (e.g., enzymes involved in oxidative phosphorylation) and the whole organismal E value.

Based on E value calculations for killifish acclimated to 5–33 °C with 5 °C intervals^36^, we predicted 12 different thermal envelopes for Northern and Southern killifish populations (Supplementary Table S3). This thermal envelope calculation allowed us to consider several thermal ranges for the E-value calculations, thus enabling us to examine potential variation in the E value along the current habitat distribution while minimizing the effects of noise to some degree. This is likely an underestimation of the number of thermal envelopes, given that we rounded the climatological minimum and maximum temperatures to the nearest 5 °C to match the acclimation data (Supplementary Table S2). Further, a major dividing zone between the Northern and Southern Atlantic Killifish subpopulations was identified at approximately around 40 N, where the Hudson River enters the Atlantic Ocean^51^. Studies also indicate a clear hybrid zone for the Northern and Southern subspecies; however, we did not account for this variation and demarcated 40 N as the dividing line between the two. To account for the different thermal physiological capacities of fish in the hybrid zone, we simulated the model using several latitudinal clinal values (39–41 N), but the final result for this region remained unchanged.

Inherent in our calculation is the assumption that killifish in a given thermal range are acclimated or locally adapted to their thermal range to optimize aerobic scope. Considering our calculations are based on lab-acclimated fish in 5 °C increments, further studies at a higher thermal resolution will help to determine more accurate thermal envelopes. Also, our model assumes that the E value of a given subpopulation is consistent across all Northern or Southern fish within the native range and remains an important area of research to explore. While these studies will increase the resolution of the habitat shift patterns, the overall predictions from our study on limited poleward movement and habitat fragmentation are likely to remain consistent.

The downscaled data from the IPCC CMIP5 project is novel and our high-resolution coastal thermal variability addresses an important gap in our understanding of future temperature variability along the North American East Coast. We used a climate data interpolation method (Delta method^52^ to downscale the coarser-resolution CMIP5 SST data (Supplementary Table S1). This method requires a higher resolution baseline climatology to add up the thin plate splined^53^ delta values. World Ocean Atlas (WOA ~ 0.25° × 0.25°^54^) climatology data is a widely used baseline climatology, but coverage along the coastal zones is insufficient. Therefore, we developed a new climatology upscaling 0.05° × 0.05° Copernicus SST data to increase the data resolution along the coastline and reduce the data source biasness. As expected, downscaled data from CMIP5 showed that SST along the killifish habitat range will be higher than its contemporary values (Fig. 4C,D and Supplementary Fig. S2) and the anomaly increases with time and the RCP scenario. Dillon et al.^27^ concluded the importance of calculating metabolic rates using high-frequency temperature data (daily temperatures) to reduce the fallacy of the averages. Here we used annual maximum and minimum SST data to calculate the location-specific metabolic rates by averaging the daily variations and the effect on the final model prediction is negligible. Our downscaled data also showed several coastal habitats that are likely to experience wide thermal fluctuations in the future compared to the contemporary period. For example, Chesapeake Bay is predicted to experience the highest SST variation during future periods as depicted in the downscaled SST product. Further, our predicted values on increased thermal variability in several important coastal habitats (e.g., the Chesapeake Bay, Southern Gulf of Maine), infer at-risk regions for inhabiting ectotherms.

SST is the only environmental parameter used in our model, while AquaMaps^55^ uses five environmental parameters (SST, sea surface salinity, sea ice content, primary productivity, and distance from the coast), and other SDMs have used a number of environmental parameters. Nonetheless, our PIBCM model can be extended to include other variables that are also determinants of MRR. Oxygen limitation from increasing oceanic hypoxic events may directly impact AS^19,20,23^ and the Metabolic Index (Φ)^28^ and mitochondrial respiration and mitochondria health index^56^ could serve as parameters to integrate changing DO and/or temperature levels into the PIBCM. Further, the inclusion of key parameters such as food availability, current fish density, and fishing pressures is critical for model accuracy and remains an important limitation in the current model.

Despite the limitations and important assumptions, our analysis on killifish presents a likely best-case scenario for coastal ectotherms and emphasizes the important role of acclimation and local adaptations that may play in determining the habitat shift patterns^10–12^. As the warming global oceans are predicted to push the species' preferred thermal envelopes northward^37^, we demonstrate that aside from strict stenotherm (e.g., some Antarctic notothenioids^57^), acclimation and local adaptions likely drive habitat fragmentations as well as aggregations, inter-subpopulation mixing, and genetic hybridizations for a coastal ectotherm. Species inhabiting lower latitudes experience habitat temperatures closer to their maximum tolerance limit, thus more likely to shift their current habitat ranges with the ocean warming (e.g., the wide habitat fragmentation predicted in this study for southern subpopulations of Atlantic killifish) than their northern conspecific species. Accordingly, certain regions of the North American coastline (e.g., Southern Gulf of Maine and Cape Cod coast) may serve as critical nursery habitats in the future while others may see significant declines in fish populations. Our PIBCM approach can estimate such habitats for economically important pelagic spices and indicate the critical need to incorporate thermal plasticity in population distribution models.

## Materials and methods

### Species-specific E value calculation

Species-specific E values were calculated using collected fish routine or standard metabolic rate data published between 2000 and 2018. Data available in the University of Maine Aquatic Science and Fisheries Abstract and Web of Science databases were used in this calculation. This study only considered the metabolic rate data of fish treated in three or more acute/acclimatory temperature treatments^58^. Metabolic rate data collected as a function of temperature, CO_2_, or Salinity were considered in the study after removing metabolic rate data collected at projected scenarios. Respective authors were contacted personally to obtain metabolic rate data unless data weren’t available online. Species-specific E values were calculated using the Arrhenius-Boltzmann^50^ (Eq. 2) relationship considering its statistical thermodynamic robustness for scaling metabolic rate variability with temperature^50^.

2 $${\mathrm{R}}_{\mathrm{b}}={\mathrm{A}}_{\mathrm{o}}{\mathrm{e}}^{-\mathrm{E}/\mathrm{KT}}$$

${R}_{b}$ is the RMR/SMR, Ao is a constant, E is the averaged activation energy (eV), K is the Boltzmann constant, and T is the temperature (Kelvin). The relationship between ln (${\mathrm{R}}_{\mathrm{b}}$) and 1/T was estimated using the least square regression method which preserves the statistical thermodynamic robustness^50^ and the slope is equal to − E/K.

### Sensitivity analysis

A sensitivity analysis was conducted to test the effect of temperature, body mass, and E value on the metabolic rate and metabolic rate range (MRR). The metabolic theory of ecology equation was used to calculate hypothetical metabolic rates for mass values ranging between 0.001 and 10 kg with 100 g intervals, E values from 0.01–1 with 0.1 intervals, and temperature values ranging between 0 and 40 °C with 5 °C intervals. 14.47 was used as the metabolic scaling component (b_o_)^29^. Metabolic rates were recalculated for the same mass and E values, but a range of temperature values between 0 and 40 °C with 2 °C intervals to estimate the effect of temperature on MRR. MRR was calculated as the difference between metabolic rates at consecutive temperatures (e.g. 0–2 °C, 2–4 °C) at a given E and mass.

### Atlantic Killifish habitat distribution data

Atlantic Killifish probability distribution data were downloaded from the AquaMaps (www.aquamaps.org). AquaMaps generates Atlantic Killifish native range probability distribution (0–1) in 0.5° × 0.5° grid resolution (Fig. 1 and Supplementary Table S1).

### Contemporary coastal Sea Surface Temperature (SST) data

**Figure 8:**
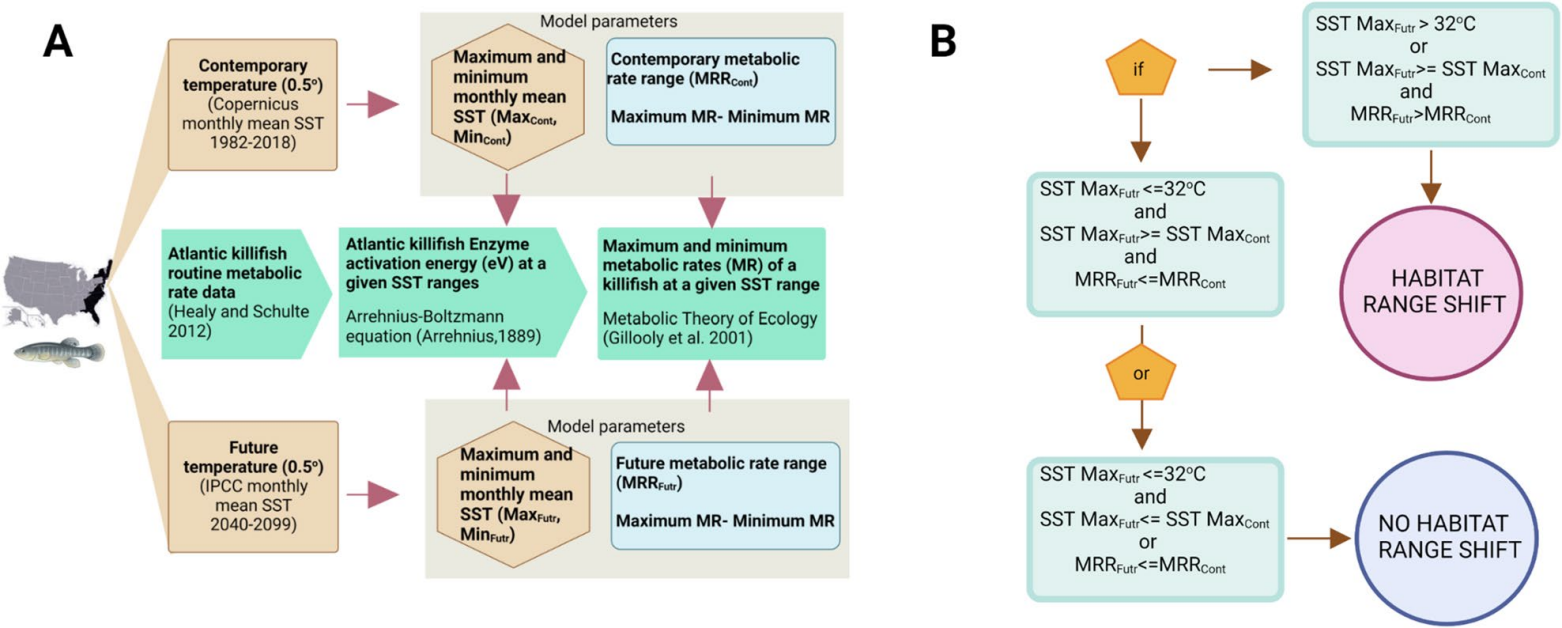
Flow chart and the model algorithm used in Physiology Integrated Bio Climate Model (PIBCM). (**A**) Flow chart of the PIBCM data processing. Pale blue, green, and orange boxes respectively represent the physiological data source, physiological theories used in the study, and the data that feed into the PIBCM algorithm. (**B**) The PIBCM model algorithm (Created with BioRender.com).

High resolution (0.05° × 0.05° × daily) reprocessed global SST data^59^ between 1982–2018 were downloaded from Marine Copernicus (https://marine.copernicus.eu) server for the model domain of 20–60 N and 100–54 W. Atlantic Killifish mostly inhabit estuaries and salt marshes^33^. Therefore, we assume that Atlantic killifish inhabits within the 0–10 m depth range and extracted SST data between 0 and 10 m depth contours using very high-resolution ETOPO2v2 bathymetry data (Supplementary Table S1) (National Geophysical Data Center) (https://www.ngdc.noaa.gov/mgg/global/relief/ETOPO2/ETOPO2v2-2006/ETOPO2v2/netCDF/). Extracted SST data were upscaled to 0.5° to match the model resolution (0.5°) (Fig. 8A). Climatological monthly maximum and minimum SST per grid point were calculated by averaging 37 years of SST data. Grid-specific SST range was calculated as the difference between the maximum and minimum climatological monthly mean SSTs.

### Downscaling the climate model projected SST data

Historical (1961–1990) and the future (the 2050s (2040–2069), and the 2080s (2070–2099)) SST data were downloaded from three climate models of the Climate Model Intercomparison Project Phase 5 (CMIP5) and the Intergovernmental Panel for Climate Change (IPCC) (Supplementary Table S1) through ESGF data distribution center at (https://esgf-node.llnl.gov/projects/cmip5/) representing three Representative Concentration Pathways (RCP 2.6,4.5, and 8.5). RCP considers three different emission scenarios, strong greenhouse gas mitigation (~ 2.6 Wm^−2^) by 2100 (RCP2.6), intermediate mitigation (~ 4.5 Wm^−2^, RCP4.5), and no mitigation (~ 8.5 Wm^−2^, RCP8.5)^56^. SST is expected to rise respectively in each RCP scenario which is ~ 1 °C relatively to 2006 in RCP2.6 and 2.4 °C compared to 2006 in RCP8.5. Most of the CMIP5 projects are based on ensemble calculations that have different initialization methods, initial states, and physical details^60^. In the CMIP5, ensemble nomenclature is based on the rip nomenclature (r—realization, i—initialization, and p-physics). Among the available ensembles, we always used the first model ensemble (r1i1p1) data in this study to reduce the complexity of the computational process.

The coarser resolution of the downloaded SST predictions (Supplementary Table S1) hindered comprehensive estimation of the future SST variability along the North American coastline. Therefore, we downscaled the individual climate model data using the Delta method^52^ to a 0.5° × 0.5° grid interval. This method takes the difference (Delta change) between the historical and future climatological SST of a given climate model, re-grid the difference to the same resolution of contemporary climatology, and adds it up to contemporary climatology. Here we developed contemporary climatology by calculating climatological monthly means of 0.5° × 0.5° gridded Copernicus SST data. Thin Plate Spline Interpolation^53^ was applied to re-grid the delta change to the contemporary climatology resolution. Delta change was calculated as the difference between the climatological monthly means of 2040–2069 (2070–2099) and the climatological monthly means of the same climate model's historical (1961–1990) period. At last, downscaled individual model SST products were averaged to get the model mean SST prediction. The final climatological monthly mean SST product was used to calculate the projected mean annual, monthly minimum, and maximum SST per grid cell.

### Metabolic Rate Range (MRR) calculation

We assumed that Atlantic Killifish in each 0.5° grid is a unique population that is locally adapted to maintain a constant MRR depending on the grid thermal envelope and the thermal envelope-specific E value. We used Atlantic Killifish routine metabolic rate (RMR) data published in Healy and Schulte^36^ to calculate the thermal envelope-specific E values. Healy and Schulte^36^, recorded RMR data of seven groups of Atlantic Killifish from both north and south subpopulations, acclimated to a temperature range of 5, 10, 15, 20, 25, 30, and 33 °C. For this, the maximum and minimum SST (current and future projected) values in each grid were rounded up to the nearest 5 °C (Supplementary Table S2). The experimental RMR values within the rounded temperature range were extracted from Healy and Schulte^36^ and plotted as a function of assay temperature to determine the slope of the regression to calculate grid-specific E value as per the Arrhenius-Boltzmann equation. The maximum and minimum metabolic rates in each grid were determined using the MTE equation and the grid-specific E value to calculate the MRR using Eq. 3 (Fig. 8A). All calculations were conducted assuming fish body weight as 10 g.

3 $$ {\text{MRR}} = {\text{maximum}}\;{\text{routine}}\;{\text{metabolic}}\;{\text{rate}} - {\text{minimum}}\;{\text{routine}}\;{\text{metabolic}}\;{\text{rate}} $$

We set the 40 N as the geographical cline between Northern and Southern Atlantic killifish populations. Therefore experimental RMR data collected using Northern Atlantic killifish subspecies were used to calculate E values in grids at or above 40 N and vice-versa. Healy and Schulte^36^ explained that both fish subpopulations reached their peak metabolic performance at 30 °C and dropped at 33 °C. Accordingly, we selected 32 °C as the physiologically optimum break temperature.

### AquaMaps model predictions for *F. heteroclitus* future distribution

AquaMaps model is regularly used as a reliable bio-climate envelope model to predict around 33,000 species’ current and future habitat distributions, and its operational process is more efficient than the other statistical or machine learning species distribution models. Therefore, we chose AquaMaps as a baseline climate envelope model results to compare with our PIBCM model results. SST climate envelopes used in AquaMaps^55^ were used to predict the Atlantic Killifish distribution in the 2050s and 2080s. AquaMaps uses a Relative Environmental Suitability (RES)^56^ method to predict species distribution considering several bio-climate indices with 5.63 °C, 7.74 °C, 21.97 °C, and 27.05 °C as the respective minimum, preferred minimum, preferred maximum, and maximum SST envelopes^6,55,61^ to predict the distribution. Climatological mean SSTs from the three downscaled RCP scenarios were used to generate habitat predictions.

### Physiology integrated bio-climate model (PIBCM) development

We assume Atlantic Killifish habitat probabilities determined (predicted probabilities by AquaMaps) in a single grid cell have been locally adapted to maintain constant MRR during the past 37 years. The fish population remains in the same location if the grid MRR in the 2050s or the 2080s is lower or equal to the historical MRR even though the grid-specific future SST exceeds its respective contemporary temperature. So, we implemented three basic model conditions to simulate Atlantic killifish future habitat ranges (Fig. 8B).

Model condition 1: if maximum SST (T_max_) ≤ 32 °C and T_max_ (future) ≥ T_max_ (contemporary) and MRR (future) ≤ MRR (contemporary) in a particular grid, the inhabited fish population stays in the same grid (no habitat range shift).

Model condition 2: if T_max_ ≤ 32 °C and T_max_ (future) ≤ T_max_ (contemporary) or MRR (future) ≤ MRR (contemporary), the inhabited fish population remains in the same grid.

Model condition 3: If T_max_ > 32 °C or T_max_ (future) ≥ T_max_ (contemporary) and MRR (future) ≥ MRR (contemporary), the inhabited fish population moves to the nearest grid location where MRR (contemporary) ≤ MRR (future).

Model building, running, and mapping (m_map toolbox v1.4^62^) were completed using the MATLAB R2022a version (University of Maine academic license 358,411). Cartesian climate data grids were re-gridded into regular latitude and longitude grids using area-weighted bilinear interpolation in Climate Data Operators (CDO v.1.9.9^63^). Fish E value calculation, statistical analysis, and thin-plate spline interpolation (fields library v.11.6^64^) were conducted in R v 4.0.3 (www.r-project.org).

### Statistical analysis

The Arrhenius-Boltzmann relationship^50^, (Eq. 2) was used to model the relationship between fish routine metabolic rates with the absolute temperature. The plot of natural logarithm transformed routine metabolic rates (ln (metabolic rate)) with the inverse temperature (T^−1^) yields a slope that is equal to − E/K where E is the enzyme activation energy, and the K is the Boltzmann constant. To estimate this relationship, we used the least-square regression (model I) in this study assuming its statistical robustness of scaling metabolic rates with independent variables such as body mass and temperature^50^. E values with probability (p) < 0.05 were considered significant.

## Supplementary Information

Supplementary Information.

## Data Availability

AquaMaps habitat probability data and temperature envelope data can be found at: https://www.aquamaps.org/. Contemporary and the IPCC CMIP5 predicted future SST data are available at the Marine Copernicus database: https://marine.copernicus.eu/ and https://esgf-node.llnl.gov/projects/cmip5/ respectively. ETOPO5 v2 bathymetry data are available at the https://www.ngdc.noaa.gov/ web link. Fish standard/routine metabolic rates are available in the main/supplementary sections of the respective manuscripts or by contacting the respective authors of each manuscript. All the processed data will be shared upon the manuscript acceptance. PIBCM code is available from the corresponding author upon reasonable request. Codes compiled to downscale CMIP5 SST data are available at the GitHub repository https://github.com/AkilaTH/CMIP5_downscaling.

## References

[1] Reygondeau G, Beaugrand G (2011). Future climate-driven shifts in distribution of Calanus finmarchicus. Glob. Change Biol..

[2] Grieve BD, Hare JA, Saba VS (2017). Projecting the effects of climate change on *Calanus finmarchicus* distribution within the U.S. Northeast Continental Shelf. Sci. Rep..

[3] Bosso L (2022). The rise and fall of an alien: Why the successful colonizer Littorina saxatilis failed to invade the Mediterranean Sea. Biol. Invasions.

[4] Guisan A, Zimmermann NE (2000). Predictive habitat distribution models in ecology. Ecol. Model..

[5] Guisan A, Thuiller W (2005). Predicting species distribution: offering more than simple habitat models. Ecol. Lett..

[6] Kaschner K, Watson R, Trites AW, Pauly D (2006). Mapping world-wide distributions of marine mammal species using a relative environmental suitability (RES) model. Mar. Ecol. Prog. Ser..

[7] Pearson RG, Dawson TP (2003). Predicting the impacts of climate change on the distribution of species: are bioclimate envelope models useful?. Glob. Ecol. Biogeogr..

[8] Buckley LB (2008). Linking traits to energetics and population dynamics to predict lizard ranges in changing environments. Am. Nat..

[9] Kolbe JJ, Kearney M, Shine R (2010). Modeling the consequences of thermal trait variation for the cane toad invasion of Australia. Ecol. Appl..

[10] Sanford E, Kelly MW (2011). Local adaptation in marine invertebrates. Ann. Rev. Mar. Sci..

[11] Somero GN, Lockwood BL, Tomanek L (2017). Biochemical Adaptation: Response to Environmental Challenges, From Life’s Origins to the Anthropocene.

[12] Kuo ES, Sanford E (2009). Geographic variation in the upper thermal limits of an intertidal snail: Implications for climate envelope models. Mar. Ecol. Prog. Ser..

[13] Smeraldo S (2018). Ignoring seasonal changes in the ecological niche of non-migratory species may lead to biases in potential distribution models: lessons from bats. Biodivers. Conserv..

[14] Gamliel I (2020). Incorporating physiology into species distribution models moderates the projected impact of warming on selected Mediterranean marine species. Ecography.

[15] Kearney MR, Wintle BA, Porter WP (2010). Correlative and mechanistic models of species distribution provide congruent forecasts under climate change. Conserv. Lett..

[16] Buckley LB, Waaser SA, MacLean HJ, Fox R (2011). Does including physiology improve species distribution model predictions of responses to recent climate change?. Ecology.

[17] Fry FEJ (1947). Effects of the environment on animal activity. Pub. Ontario Fish. Lab. No. 68. Toronto Studies Biol. Ser..

[18] Brett JR (1971). Energetic responses of salmon to temperature. A study of some thermal relations in the physiology and freshwater ecology of sockeye salmon (*Oncorhynchus nerkd*). Am Zoologist.

[19] Pörtner HO, Knust R (2007). Climate change affects marine fishes through the oxygen limitation of thermal tolerance. Science.

[20] Pörtner HO, Farrell AP (2008). Physiology and climate change. Science.

[21] Eliason EJ (2011). Differences in thermal tolerance among sockeye salmon populations. Science.

[22] Donelson JM, Munday PL, McCormick MI, Pitcher CR (2012). Rapid transgenerational acclimation of a tropical reef fish to climate change. Nat. Clim. Change.

[23] Pörtner H (2001). Climate change and temperature-dependent biogeography: Oxygen limitation of thermal tolerance in animals. Naturwissenschaften.

[24] Pörtner H-O (2010). Oxygen-and capacity-limitation of thermal tolerance: A matrix for integrating climate-related stressor effects in marine ecosystems. J. Exp. Biol..

[25] Clark TD, Sandblom E, Jutfelt F (2013). Response to Farrell and to Pörtner and Giomi. J. Exp. Biol..

[26] Farrell AP (2013). Aerobic scope and its optimum temperature: Clarifying their usefulness and limitations: Correspondence on J. Exp. Biol. 216, 2771–2782. J. Exp. Biol..

[27] Dillon ME, Wang G, Huey RB (2010). Global metabolic impacts of recent climate warming. Nature.

[28] Deutsch C, Ferrel A, Seibel B, Pörtner H-O, Huey RB (2015). Climate change tightens a metabolic constraint on marine habitats. Science.

[29] Gillooly JF, Brown JH, West GB, Savage VM, Charnov EL (2001). Effects of size and temperature on metabolic rate. Science.

[30] Brown JH, Gillooly JF, Allen AP, Savage VM, West GB (2004). Toward a metabolic theory of ecology. Ecology.

[31] Clarke A (2004). Is there a universal temperature dependence of metabolism?. Funct. Ecol..

[32] Clarke A, Fraser KPP (2004). Why does metabolism scale with temperature?. Funct. Ecol..

[33] Fangue NA, Hofmeister M, Schulte PM (2006). Intraspecific variation in thermal tolerance and heat shock protein gene expression in common killifish, *Fundulus heteroclitus*. J. Exp. Biol..

[34] Dhillon RS, Schulte PM (2011). Intraspecific variation in the thermal plasticity of mitochondria in killifish. J. Exp. Biol..

[35] Fangue NA, Podrabsky JE, Crawshaw LI, Schulte PM (2009). Countergradient variation in temperature preference in populations of killifish *Fundulus heteroclitus*. Physiol. Biochem. Zool..

[36] Healy TM, Schulte PM (2012). Thermal acclimation is not necessary to maintain a wide thermal breadth of aerobic scope in the common killifish (*Fundulus heteroclitus*). Physiol. Biochem. Zool..

[37] Chust G (2014). Are *Calanus* spp. shifting poleward in the North Atlantic? A habitat modelling approach. ICES J. Mar. Sci..

[38] Norin T, Malte H, Clark TD (2014). Aerobic scope does not predict the performance of a tropical eurythermal fish at elevated temperatures. J. Exp. Biol..

[39] Payne NL (2016). Temperature dependence of fish performance in the wild: links with species biogeography and physiological thermal tolerance. Funct. Ecol..

[40] Raffel TR (2013). Disease and thermal acclimation in a more variable and unpredictable climate. Nat. Clim. Change.

[41] Sinclair BJ (2016). Can we predict ectotherm responses to climate change using thermal performance curves and body temperatures?. Ecol. Lett..

[42] Dahlke FT (2018). Northern cod species face spawning habitat losses if global warming exceeds 1.5°C. Sci. Adv..

[43] Pörtner H-O, Giomi F (2013). Nothing in experimental biology makes sense except in the light of ecology and evolution: Correspondence on J. Exp. Biol. 2771-2782. J. Exp. Biol..

[44] Pörtner H-O (2014). How and how not to investigate the oxygen and capacity limitation of thermal tolerance (OCLTT) and aerobic scope: Remarks on the article by Gräns et al. J. Exp. Biol..

[45] Kleiber M (1932). Body size and metabolism. Hilgardia.

[46] Killen SS, Atkinson D, Glazier DS (2010). The intraspecific scaling of metabolic rate with body mass in fishes depends on lifestyle and temperature. Ecol. Lett..

[47] Norin T, Gamperl AK (2018). Metabolic scaling of individuals vs. populations: Evidence for variation in scaling exponents at different hierarchical levels. Funct. Ecol..

[48] Jayasundara N, Kozal JS, Arnold MC, Chan SSL, Giulio RTD (2015). High-throughput tissue bioenergetics analysis reveals identical metabolic allometric scaling for teleost hearts and whole organisms. PLoS ONE.

[49] Kinnison MT, Unwin MJ, Quinn TP (2003). Migratory costs and contemporary evolution of reproductive allocation in male chinook salmon. J. Evol. Biol..

[50] Clarke A, Johnston NM (1999). Scaling of metabolic rate with body mass and temperature in teleost fish. J. Anim. Ecol..

[51] Duvernell DD, Lindmeier JB, Faust KE, Whitehead A (2008). Relative influences of historical and contemporary forces shaping the distribution of genetic variation in the Atlantic killifish, *Fundulus heteroclitus*. Mol. Ecol..

[52] Navarro-Racines C, Tarapues J, Thornton P, Jarvis A, Ramirez-Villegas J (2020). High-resolution and bias-corrected CMIP5 projections for climate change impact assessments. Sci. Data.

[53] Franke R (1982). Scattered data interpolation: Tests of some methods. Math. Comput..

[54] Levitus S (2012). World ocean heat content and thermosteric sea level change (0–2000 m), 1955–2010. Geophys. Res. Lett..

[55] Kaschner K (2019). AquaMaps: Predicted Range Maps for Aquatic Species.

[56] Jayasundara N (2017). Ecological significance of mitochondrial toxicants. Toxicology.

[57] Beers JM, Jayasundara N (2015). Antarctic notothenioid fish: what are the future consequences of ‘losses’ and ‘gains’ acquired during long-term evolution at cold and stable temperatures?. J. Exp. Biol..

[58] Lear KO (2019). Thermal performance responses in free-ranging elasmobranchs depend on habitat use and body size. Oecologia.

[59] Good S (2020). The current configuration of the OSTIA system for operational production of foundation sea surface temperature and ice concentration analyses. Remote Sens..

[60] Stocker T (2014). Climate Change 2013: The Physical Science Basis: Working Group I Contribution to the Fifth Assessment Report of the Intergovernmental Panel on Climate Change.

[61] Ready J (2010). Predicting the distributions of marine organisms at the global scale. Ecol. Model..

[62] Pawlowicz, R. *M_Map: A Mapping Package for MATLAB, Version 1.4 m. Computer Software, UBC EOAS*. https://www.eoas.ubc.ca/rich/map.html (2020).

[63] Schulzweida, U., Kornblueh, L. & Quast, R*. CDO User’s Guide. Climate Data Operators, Version* **1**, (2006).

[64] Nychka, D., Furrer, R., Paige, J. & Sain, S. *Fields: Tools for Spatial Data. R Package Version 11.6*. (2017).

[65] Chen Z, Farrell AP, Matala A, Narum SR (2018). Mechanisms of thermal adaptation and evolutionary potential of conspecific populations to changing environments. Mol. Ecol..

[66] da Silva CRB, Riginos C, Wilson RS (2019). An intertidal fish shows thermal acclimation despite living in a rapidly fluctuating environment. J. Comp. Physiol. B..

[67] Slesinger E (2019). The effect of ocean warming on black sea bass (Centropristis striata) aerobic scope and hypoxia tolerance. PLoS ONE.

[68] Moffett ER, Fryxell DC, Palkovacs EP, Kinnison MT, Simon KS (2018). Local adaptation reduces the metabolic cost of environmental warming. Ecology.

[69] Turker H (2011). The effect of water temperature on standard and routine metabolic rate in two different sizes of Nile tilapia. Kafkas Universitesi Veteriner Fakultesi Dergisi.

[70] Hvas M, Folkedal O, Imsland A, Oppedal F (2017). The effect of thermal acclimation on aerobic scope and critical swimming speed in Atlantic salmon, Salmo salar. J. Exp. Biol..

[71] Ohlberger J, Mehner T, Staaks G, Hölker F (2012). Intraspecific temperature dependence of the scaling of metabolic rate with body mass in fishes and its ecological implications. Oikos.

[72] Kunz KL (2016). New encounters in Arctic waters: A comparison of metabolism and performance of polar cod (*Boreogadus saida*) and Atlantic cod (*Gadus morhua*) under ocean acidification and warming. Polar Biol..

[73] Norin T, Bailey JA, Gamperl AK (2019). Thermal biology and swimming performance of Atlantic cod (Gadus morhua) and haddock (Melanogrammus aeglefinus). PeerJ.

[74] Nowell LB (2015). Swimming energetics and thermal ecology of adult bonefish (Albula vulpes): A combined laboratory and field study in Eleuthera, The Bahamas. Environ. Biol. Fishes.

[75] Pang X, Yuan X-Z, Cao Z-D, Zhang Y-G, Fu S-J (2015). The effect of temperature on repeat swimming performance in juvenile qingbo (Spinibarbus sinensis). Fish Physiol. Biochem..

[76] Schwieterman GD (2019). Metabolic Rates and Hypoxia Tolerences of clearnose skate (*Rostaraja eglanteria*), summer flounder (*Paralichthys dentatus*), and thorny skate (*Amblyraja radiata*). Biology.

[77] Xie H (2017). Effects of acute temperature change and temperature acclimation on the respiratory metabolism of the snakehead. Turk. J. Fish. Aquat. Sci..

