# Supplementary material for: Thermal physiology integrated species distribution model predicts profound habitat fragmentation for estuarine fish with ocean warming
Source: Sci Rep. 2022 Dec 16;12:21781. doi: 10.1038/s41598-022-25419-4 (PMC9758224; doi:10.1038/s41598-022-25419-4)
Supplement: Supplementary file 1 — Supplementary Information. [file 41598_2022_25419_MOESM1_ESM.pdf]

Thermal Physiology integrated species distribution model predicts profound habitat fragmentation for estuarine fish with ocean warming

Akila Harishchandra, Huijie Xue, Santiago Salinas, Nishad Jayasundara

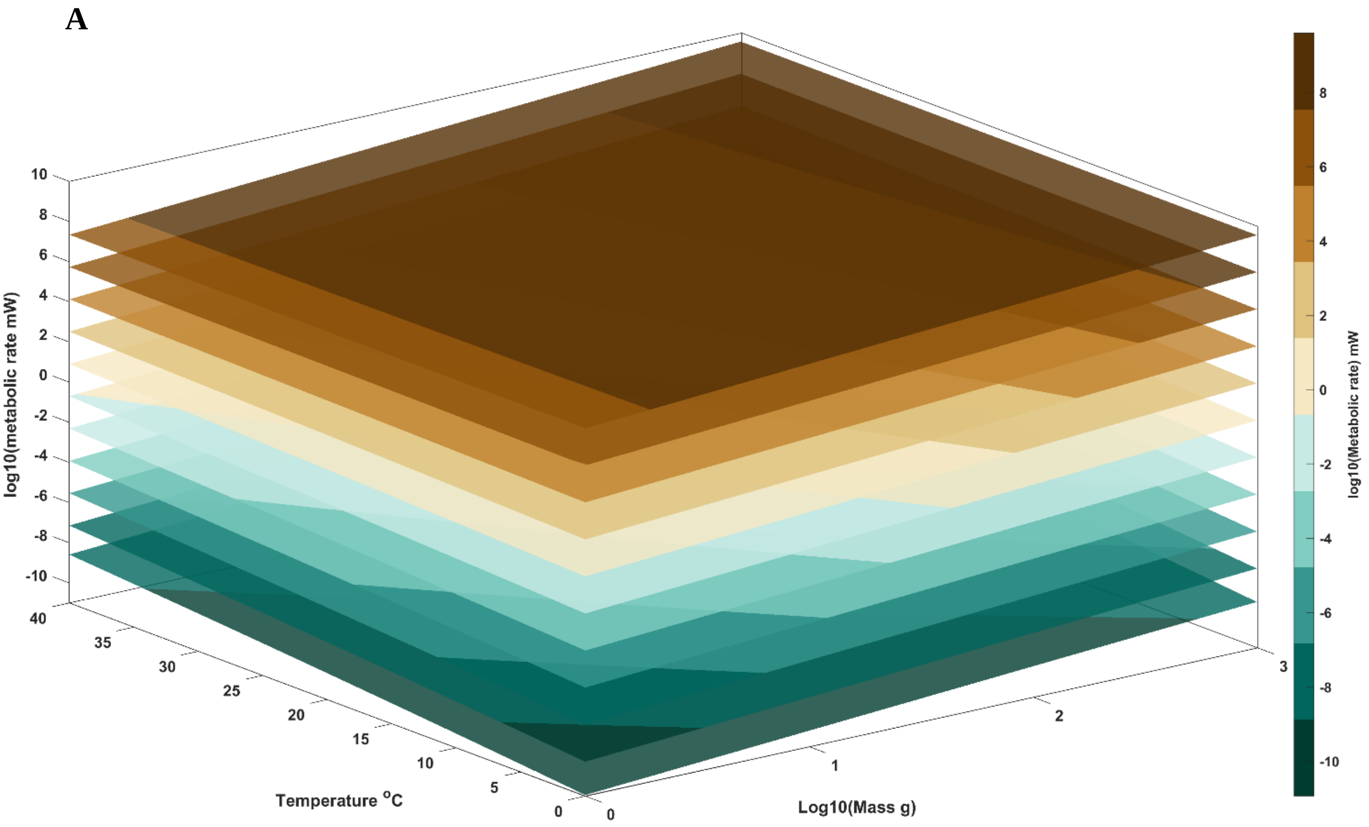

Fig.S1. Sensitivity analysis calculates hypothetical metabolic rates (mW) for a range of temperature (0-40 ℃), body mass (1-1000g), and E values (0.01-1eV) using the Metabolic Theory of Ecology equation. (A) Metabolic rates that are calculated for each temperature, mass, and E value (B) Metabolic rate (Log10) changes as a function of body mass (metabolic rates were calculated at 20℃ and E values from 0.01 to 1 eV) (C) Metabolic rate (Log10) changes as a function of temperature (metabolic rates were calculated at 100g and the full E value range) (D) metabolic rate range ((the difference between the metabolic rates calculated at consecutive temperatures) changes with the respective temperatures. Different color lines in figures B, C, and D indicate the respective E value (displayed in the legend box) used for the calculation.

B

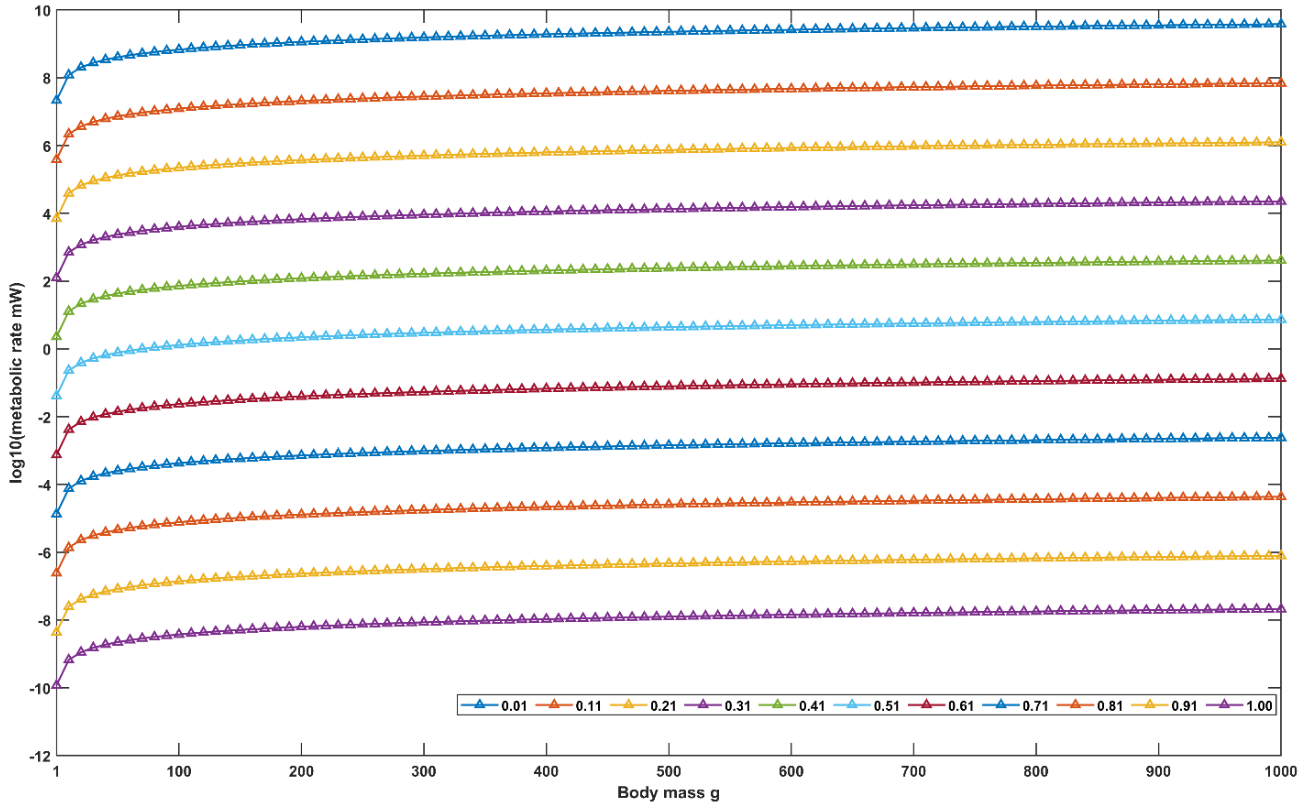

Fig.S1. Sensitivity analysis calculates hypothetical metabolic rates (mW) for a range of temperature (0-40 ℃), body mass (1-1000g), and E values (0.01-1eV) using the Metabolic Theory of Ecology equation. (A) Metabolic rates that are calculated for each temperature, mass, and E value (B) Metabolic rate (Log10) changes as a function of body mass (metabolic rates were calculated at 20℃ and E values from 0.01 to 1 eV) (C) Metabolic rate (Log10) changes as a function of temperature (metabolic rates were calculated at 100g and the full E value range) (D) metabolic rate range ((the difference between the metabolic rates calculated at consecutive temperatures) changes with the respective temperatures. Different color lines in figures B, C, and D indicate the respective E value (displayed in the legend box) used for the calculation.

C

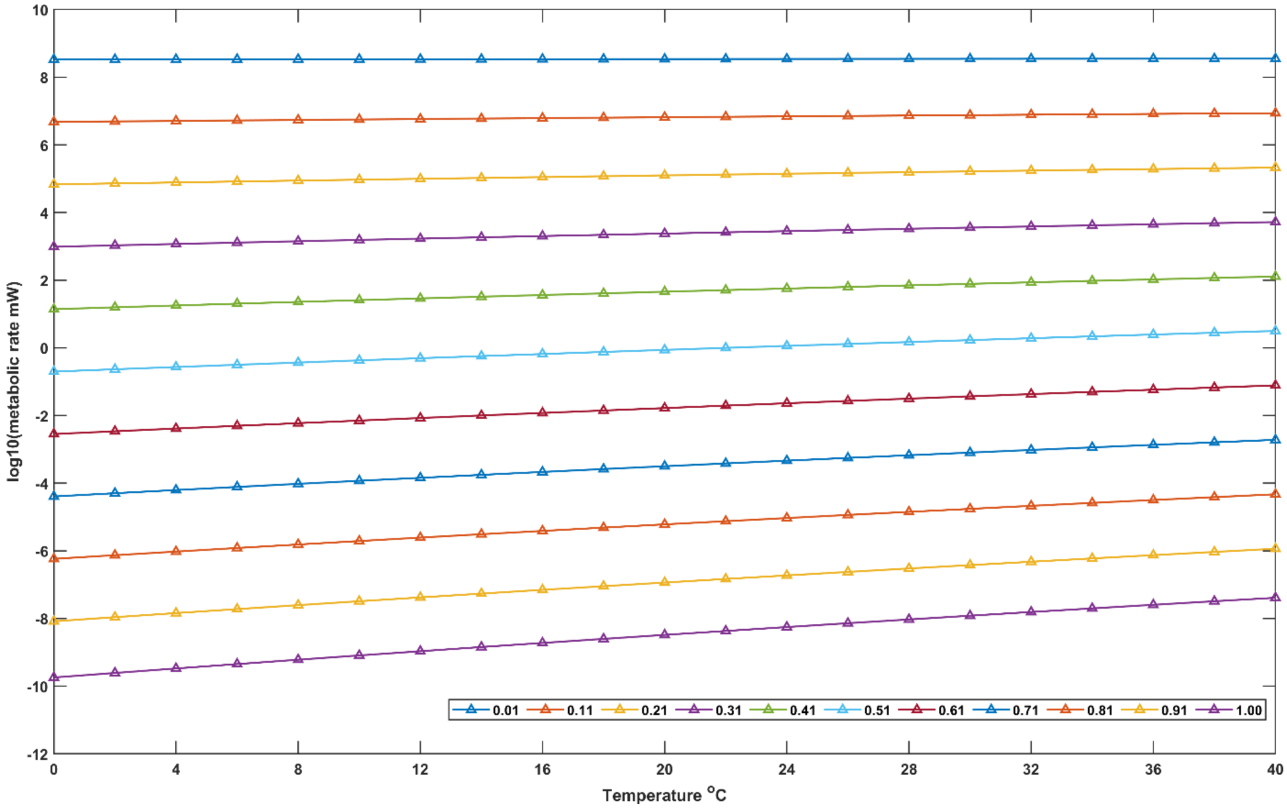

Fig.S1. Sensitivity analysis calculates hypothetical metabolic rates (mW) for a range of temperature (0-40 ℃), body mass (1-1000g), and E values (0.01-1eV) using the Metabolic Theory of Ecology equation. (A) Metabolic rates that are calculated for each temperature, mass, and E value (B) Metabolic rate (Log10) changes as a function of body mass (metabolic rates were calculated at 20℃ and E values from 0.01 to 1 eV) (C) Metabolic rate (Log10) changes as a function of temperature (metabolic rates were calculated at 100g and the full E value range) (D) metabolic rate range ((the difference between the metabolic rates calculated at consecutive temperatures) changes with the respective temperatures. Different color lines in figures B, C, and D indicate the respective E value (displayed in the legend box) used for the calculation.

D

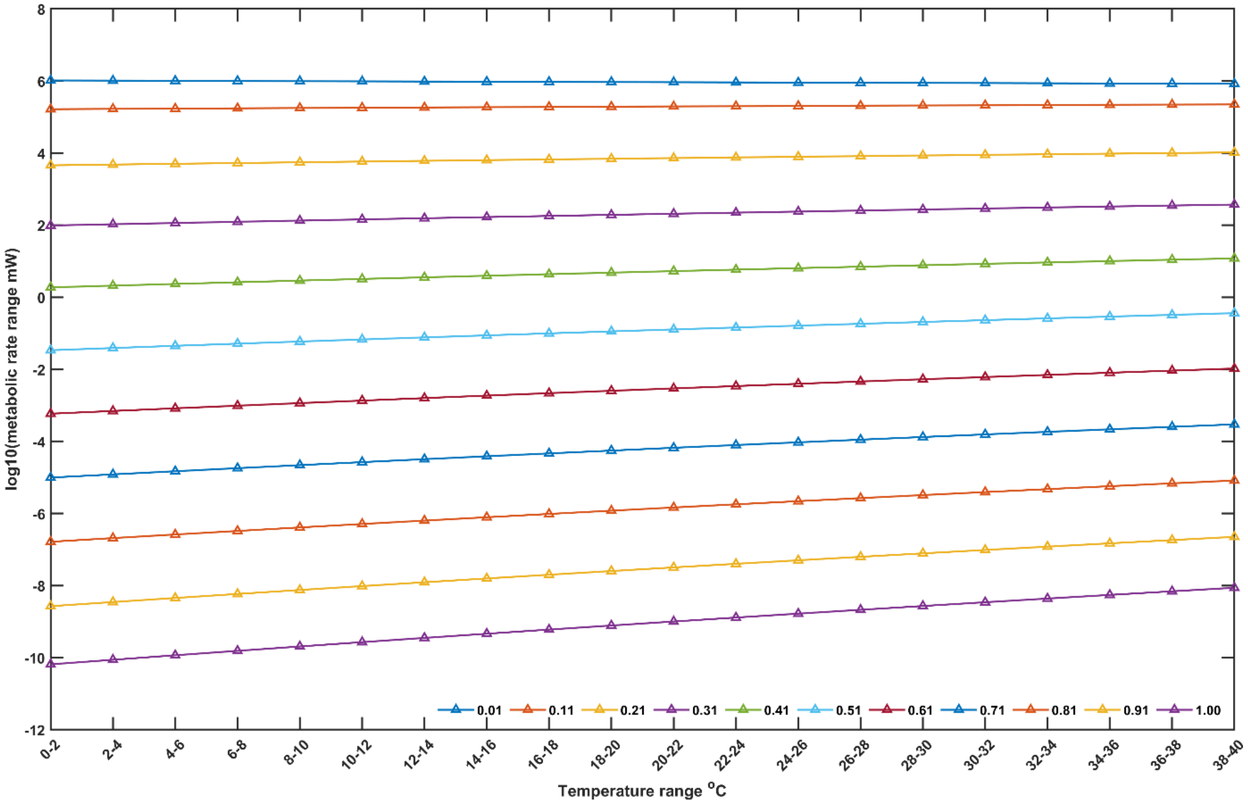

Fig.S1. Sensitivity analysis calculates hypothetical metabolic rates (mW) for a range of temperature (0-40 ℃), body mass (1-1000g), and E values (0.01-1eV) using the Metabolic Theory of Ecology equation. (A) Metabolic rates that are calculated for each temperature, mass, and E value (B) Metabolic rate (Log10) changes as a function of body mass (metabolic rates were calculated at 20℃ and E values from 0.01 to 1 eV) (C) Metabolic rate (Log10) changes as a function of temperature (metabolic rates were calculated at 100g and the full E value range) (D) metabolic rate range ((the difference between the metabolic rates calculated at consecutive temperatures) changes with the respective temperatures. Different color lines in figures B, C, and D indicate the respective E value (displayed in the legend box) used for the calculation.

A

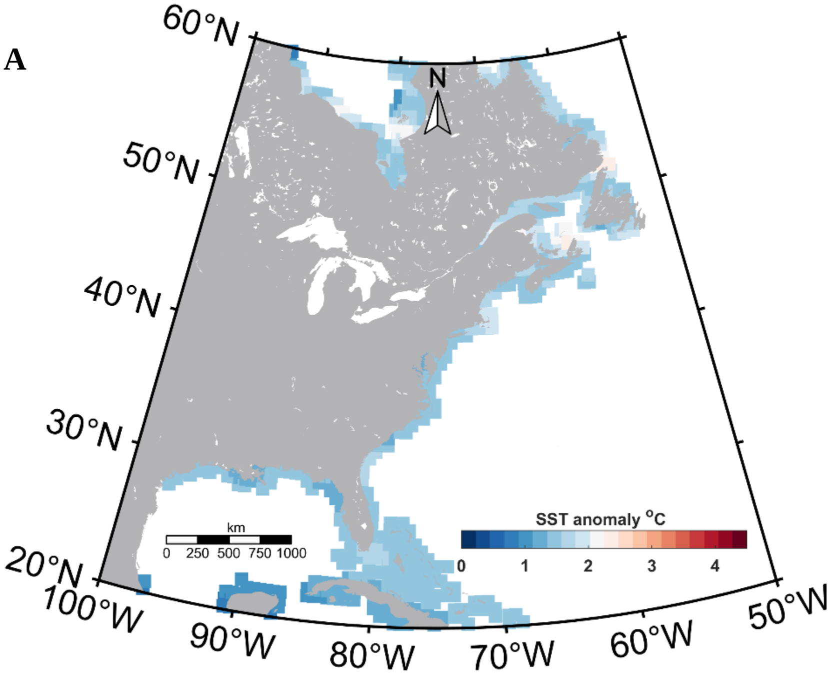

Fig. S2. Projected Sea surface temperature (SST) anomaly along the East coast of North America in the 2050s and 2080s derived using different emission scenarios. (A)SST anomaly (the difference between the climatological mean SST in the contemporary period (1982-2018) and the future periods) in the 2050s, which is predicted under Representative Concentration Pathways (RCPs) 4.5, (B) RCP8.5, (C) SST anomaly in the 2080s, RCP2.6 and (D) RCP4.5

B

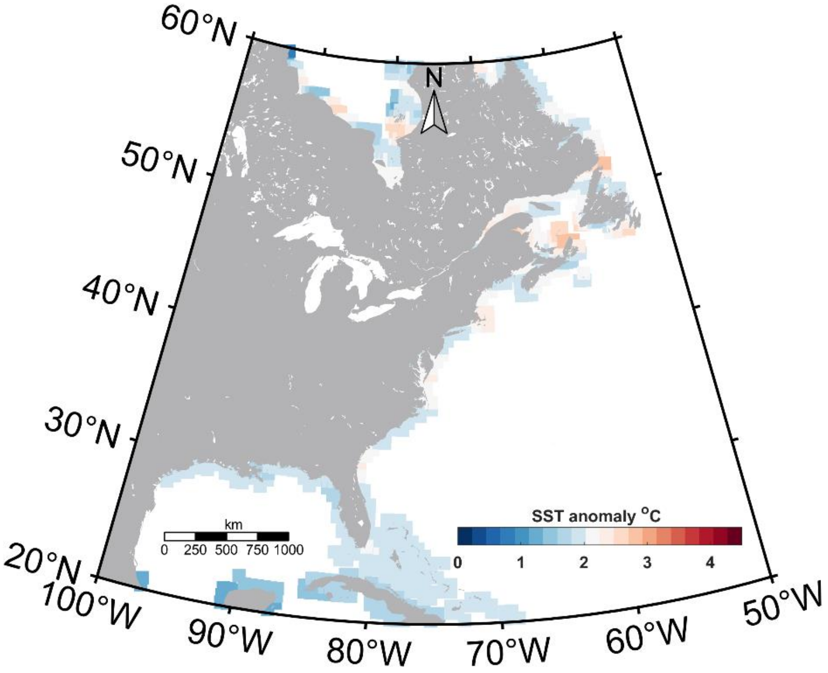

Fig. S2. Projected Sea surface temperature (SST) anomaly along the East coast of North America in the 2050s and 2080s derived using different emission scenarios. (A)SST anomaly (the difference between the climatological mean SST in the contemporary period (1982-2018) and the future periods) in the 2050s, which is predicted under Representative Concentration Pathways (RCPs) 4.5, (B) RCP8.5, (C) SST anomaly in the 2080s, RCP2.6 and (D) RCP4.5

C

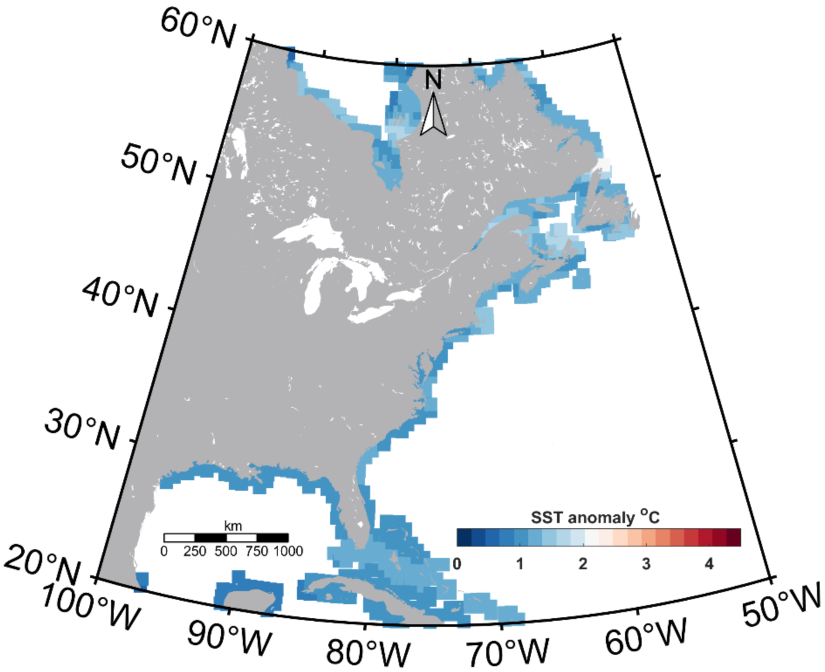

Fig. S2. Projected Sea surface temperature (SST) anomaly along the East coast of North America in the 2050s and 2080s derived using different emission scenarios. (A)SST anomaly (the difference between the climatological mean SST in the contemporary period (1982-2018) and the future periods) in the 2050s, which is predicted under Representative Concentration Pathways (RCPs) 4.5, (B) RCP8.5, (C) SST anomaly in the 2080s, RCP2.6 and (D) RCP4.5

D

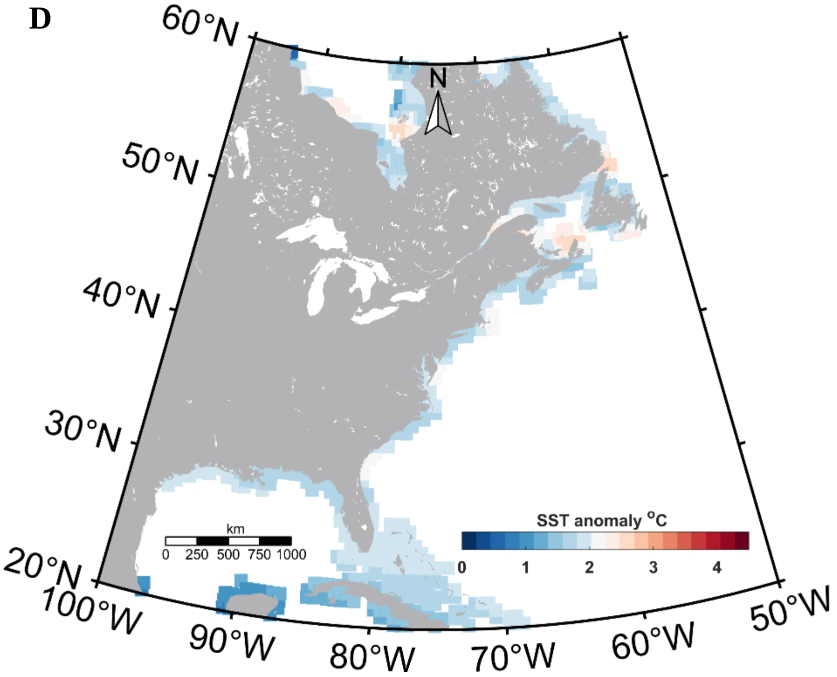

Fig. S2. Projected Sea surface temperature (SST) anomaly along the East coast of North America in the 2050s and 2080s derived using different emission scenarios. (A)SST anomaly (the difference between the climatological mean SST in the contemporary period (1982-2018) and the future periods) in the 2050s, which is predicted under Representative Concentration Pathways (RCPs) 4.5, (B) RCP8.5, (C) SST anomaly in the 2080s, RCP2.6 and (D) RCP4.5

A

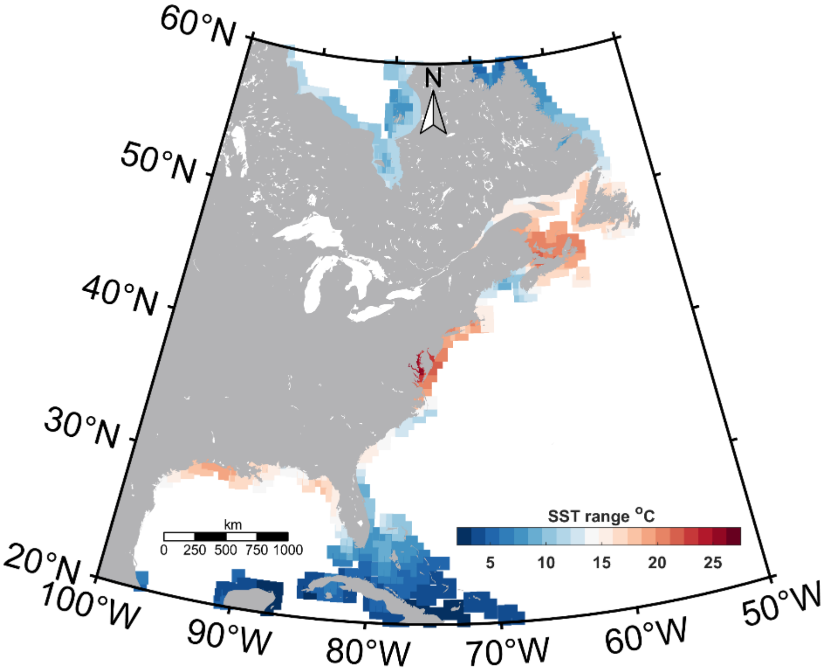

Fig. S3. Projected Sea surface temperature (SST) range along the East Coast of North America in the 2050s and 2080s derived using different emission scenarios. (A)SST range (the difference between the climatological maximum and minimum SST at a given period) in the 2050s, which is predicted under Representative Concentration Pathways (RCPs) 4.5, (B) RCP8.5, (C) SST range in the 2080s, RCP2.6 and (D) RCP4.5

B

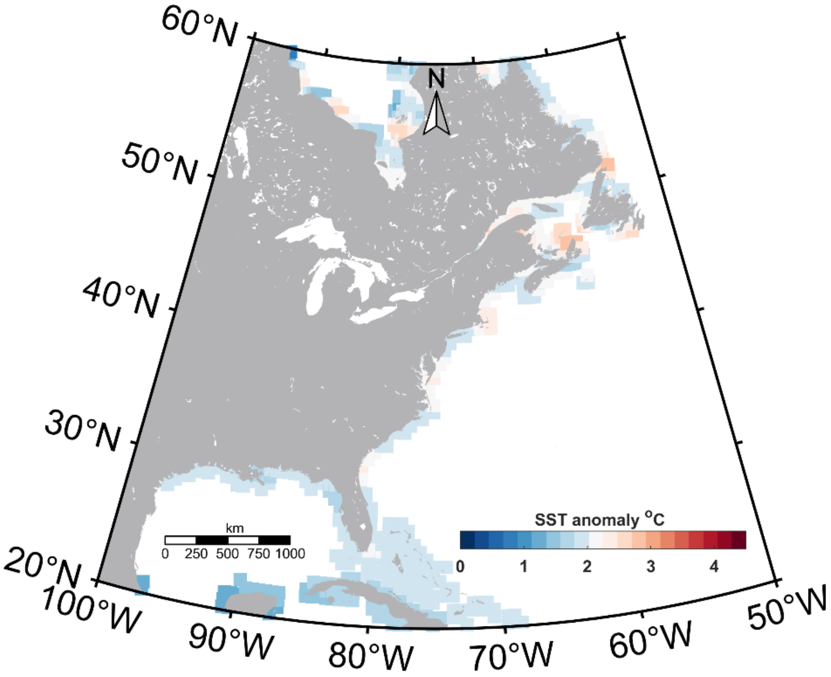

Fig. S3. Projected Sea surface temperature (SST) range along the East Coast of North America in the 2050s and 2080s derived using different emission scenarios. (A)SST range (the difference between the climatological maximum and minimum SST at a given period) in the 2050s, which is predicted under Representative Concentration Pathways (RCPs) 4.5, (B) RCP8.5, (C) SST range in the 2080s, RCP2.6 and (D) RCP4.5

C

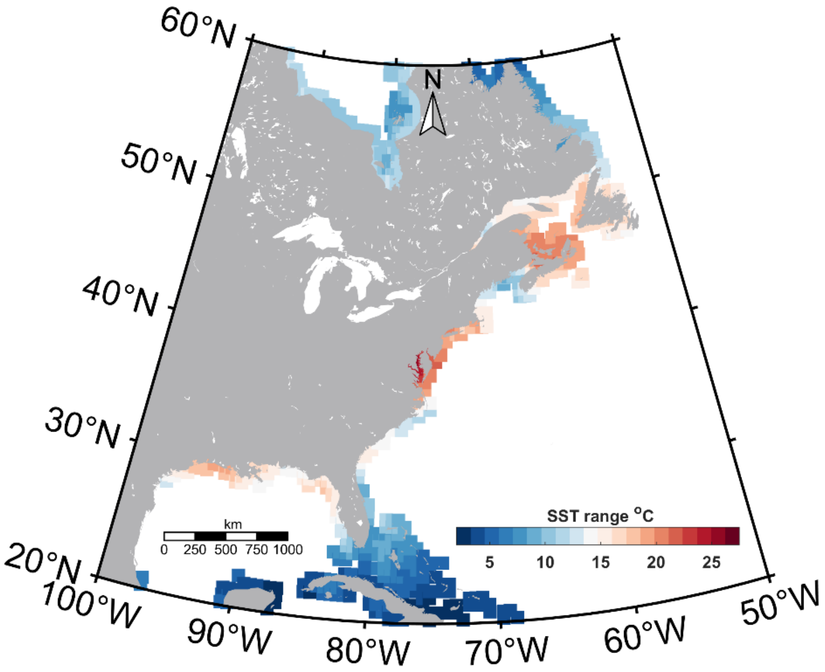

Fig. S3. Projected Sea surface temperature (SST) range along the East Coast of North America in the 2050s and 2080s derived using different emission scenarios. (A)SST range (the difference between the climatological maximum and minimum SST at a given period) in the 2050s, which is predicted under Representative Concentration Pathways (RCPs) 4.5, (B) RCP8.5, (C) SST range in the 2080s, RCP2.6 and (D) RCP4.5

D

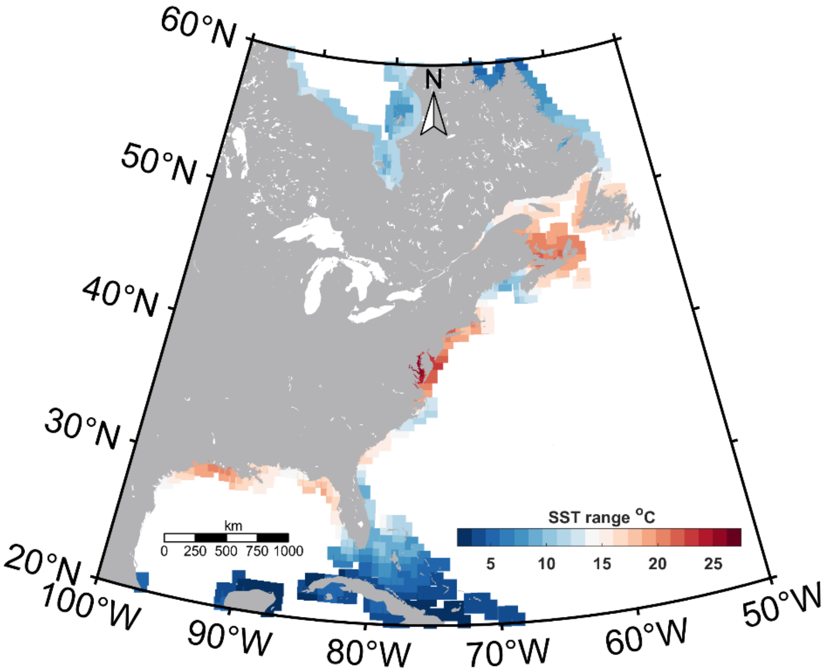

Fig. S3. Projected Sea surface temperature (SST) range along the East Coast of North America in the 2050s and 2080s derived using different emission scenarios. (A)SST range (the difference between the climatological maximum and minimum SST at a given period) in the 2050s, which is predicted under Representative Concentration Pathways (RCPs) 4.5, (B) RCP8.5, (C) SST range in the 2080s, RCP2.6 and (D) RCP4.5

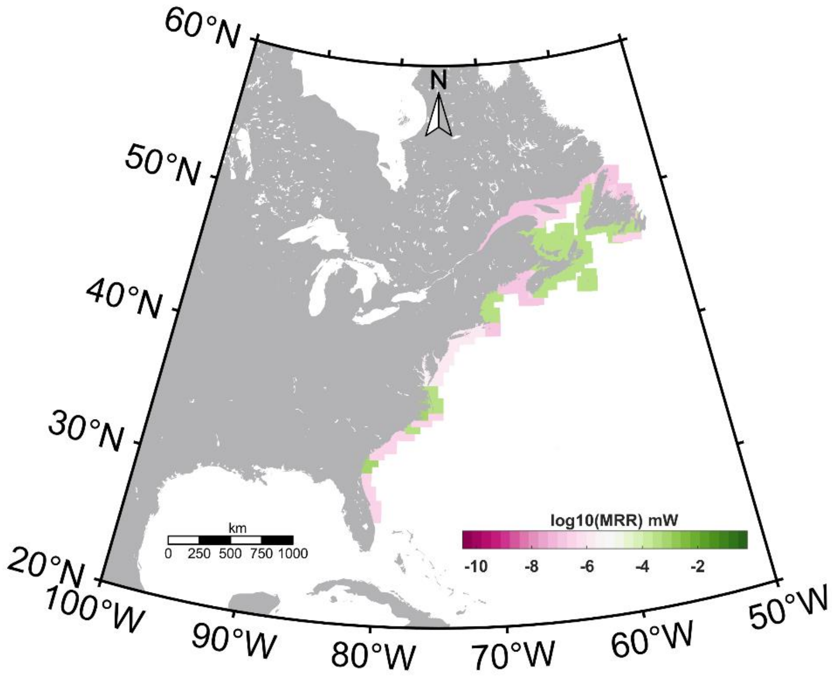

Fig. S4. Metabolic rate range (MRR) (the difference between the maximum and minimum routine metabolic rates of a given grid cell) along the Atlantic killifish native habitat range during the contemporary period (1982-2018).

A

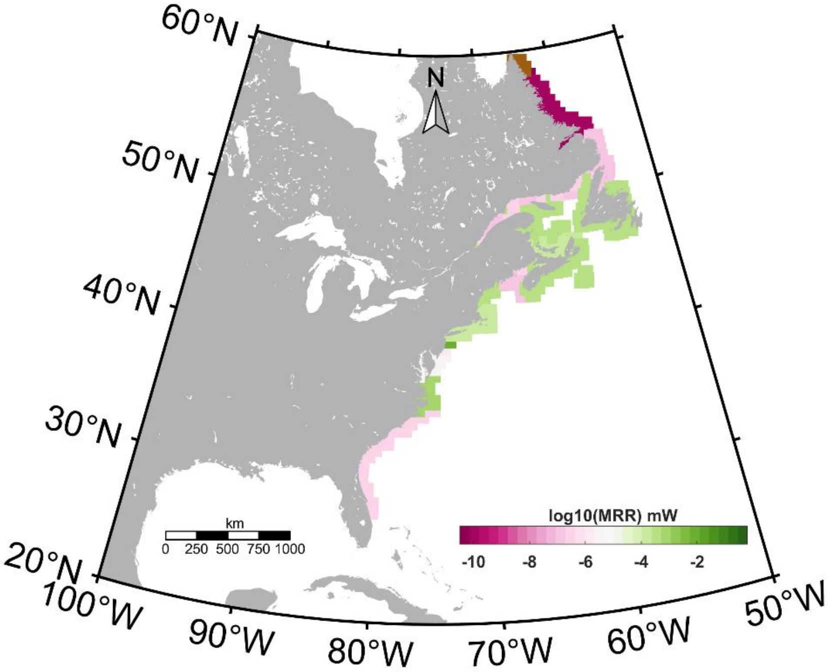

Fig. S5. Latitudinal metabolic rate range (MRR) variability along the Northeast American coastal zone. A-C, MRR in the 2050s calculated based on Representative Concentration Pathways (RCP) 2.6,4.5 and 8.5 projected sea surface temperature data, respectively, and D-F, for the 2080s. The legend shows the grids that exceeded the physiological break temperature for Atlantic killifish (32℃) we used in the model.

B

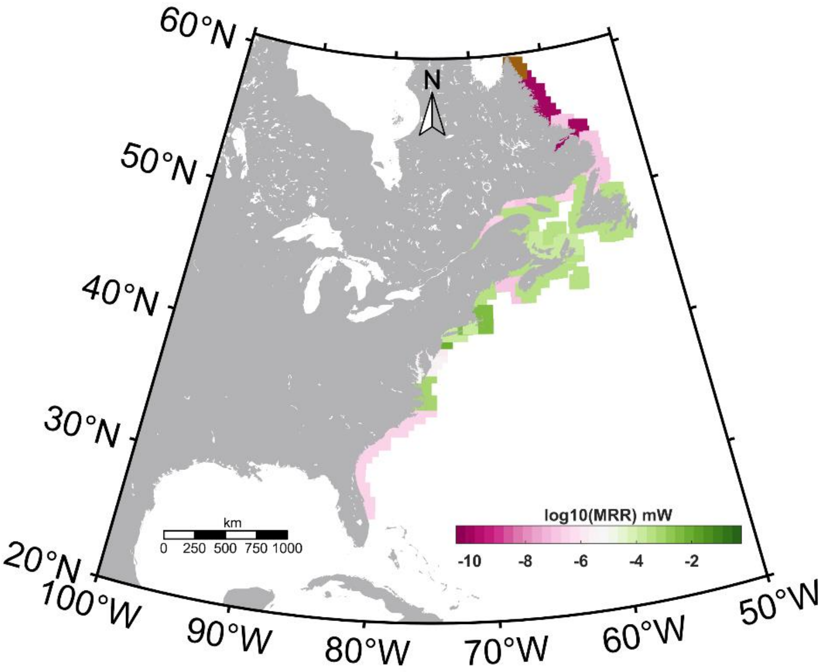

Fig. S5. Latitudinal metabolic rate range (MRR) variability along the Northeast American coastal zone. A-C, MRR in the 2050s calculated based on Representative Concentration Pathways (RCP) 2.6,4.5 and 8.5 projected sea surface temperature data, respectively, and D-F, for the 2080s. The legend shows the grids that exceeded the physiological break temperature for Atlantic killifish (32℃) we used in the model.

C

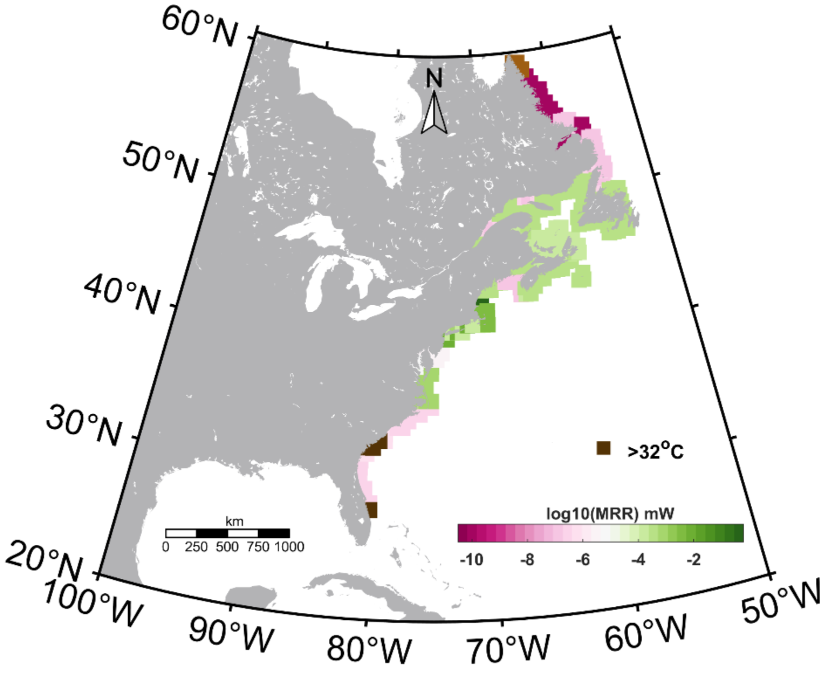

Fig. S5. Latitudinal metabolic rate range (MRR) variability along the Northeast American coastal zone. A-C, MRR in the 2050s calculated based on Representative Concentration Pathways (RCP) 2.6,4.5 and 8.5 projected sea surface temperature data, respectively, and D-F, for the 2080s. The legend shows the grids that exceeded the physiological break temperature for Atlantic killifish (32℃) we used in the model.

D

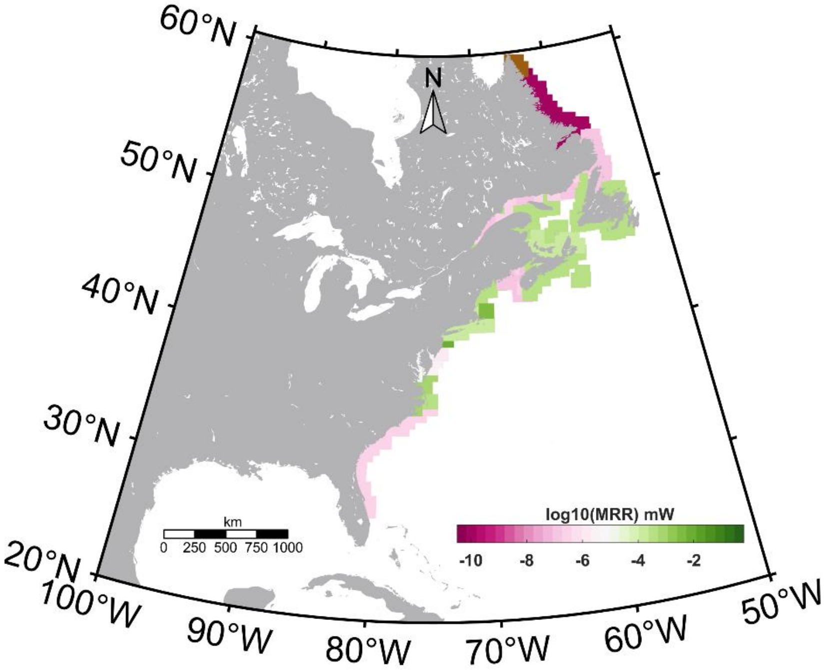

Fig. S5. Latitudinal metabolic rate range (MRR) variability along the Northeast American coastal zone. A-C, MRR in the 2050s calculated based on Representative Concentration Pathways (RCP) 2.6,4.5 and 8.5 projected sea surface temperature data, respectively, and D-F, for the 2080s. The legend shows the grids that exceeded the physiological break temperature for Atlantic killifish (32℃) we used in the model.

E

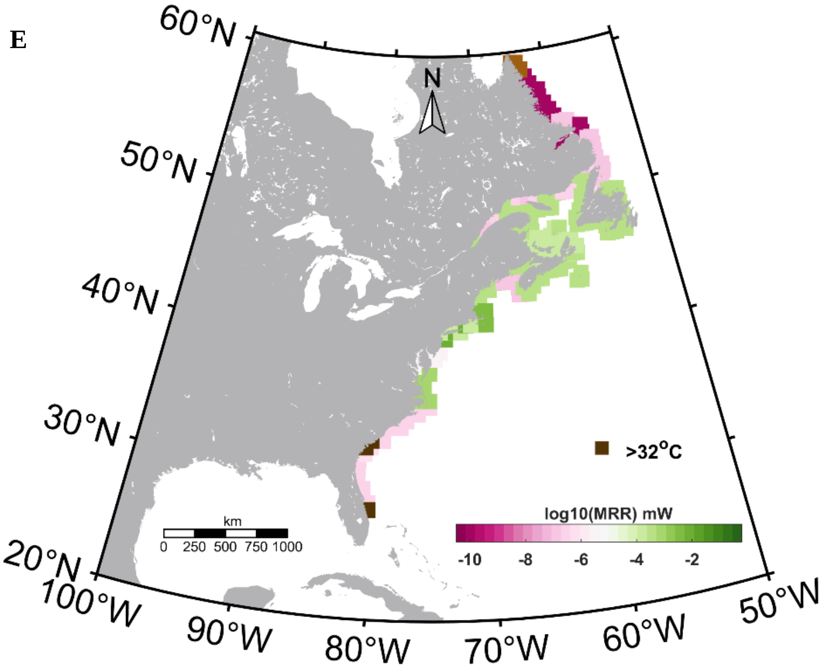

Fig. S5. Latitudinal metabolic rate range (MRR) variability along the Northeast American coastal zone. A-C, MRR in the 2050s calculated based on Representative Concentration Pathways (RCP) 2.6,4.5 and 8.5 projected sea surface temperature data, respectively, and D-F, for the 2080s. The legend shows the grids that exceeded the physiological break temperature for Atlantic killifish (32℃) we used in the model.

F

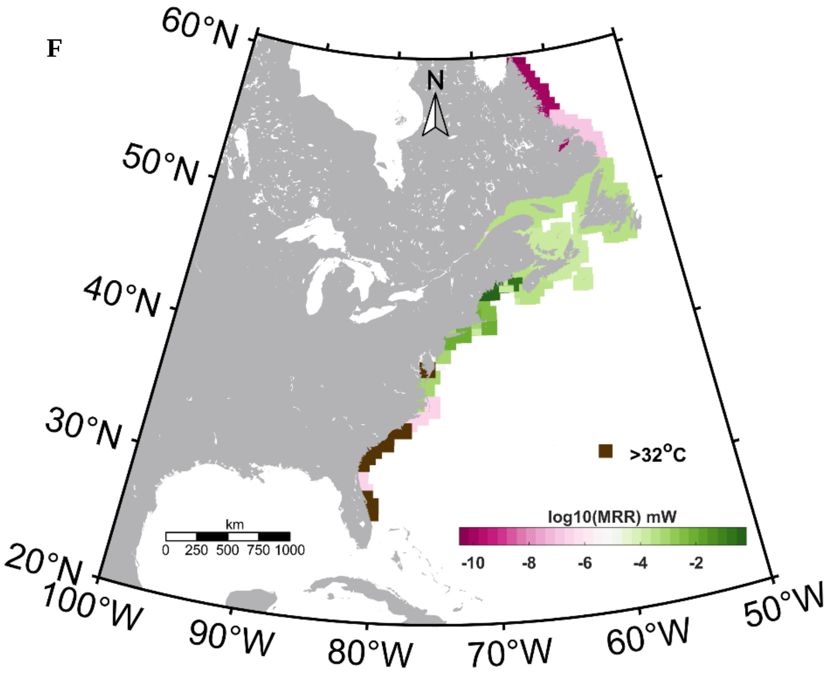

Fig. S5. Latitudinal metabolic rate range (MRR) variability along the Northeast American coastal zone. A-C, MRR in the 2050s calculated based on Representative Concentration Pathways (RCP) 2.6,4.5 and 8.5 projected sea surface temperature data, respectively, and D-F, for the 2080s. The legend shows the grids that exceeded the physiological break temperature for Atlantic killifish (32℃) we used in the model.

A

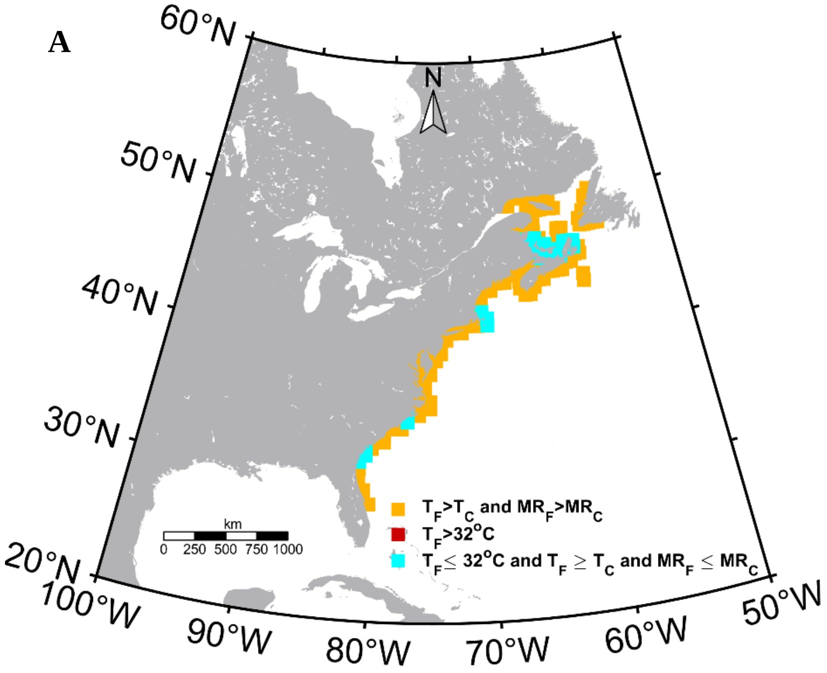

Fig. S6. Atlantic killifish populations pooled under the conditions implemented in the physiology integrated bio-climate model (PIBCM). A-C, Atlantic killifish populations pooled under PIBCM conditions in the 2050s based on data from Representative Concentration Pathways (RCPs) 2.6,4.5 and 8.5, respectively. D-F, Atlantic killifish populations pooled under PIBCM conditions in the 2080s under the same respective RCPs. In the figure legend, T is SST, F is future, C is contemporary, MR is metabolic rate range

B

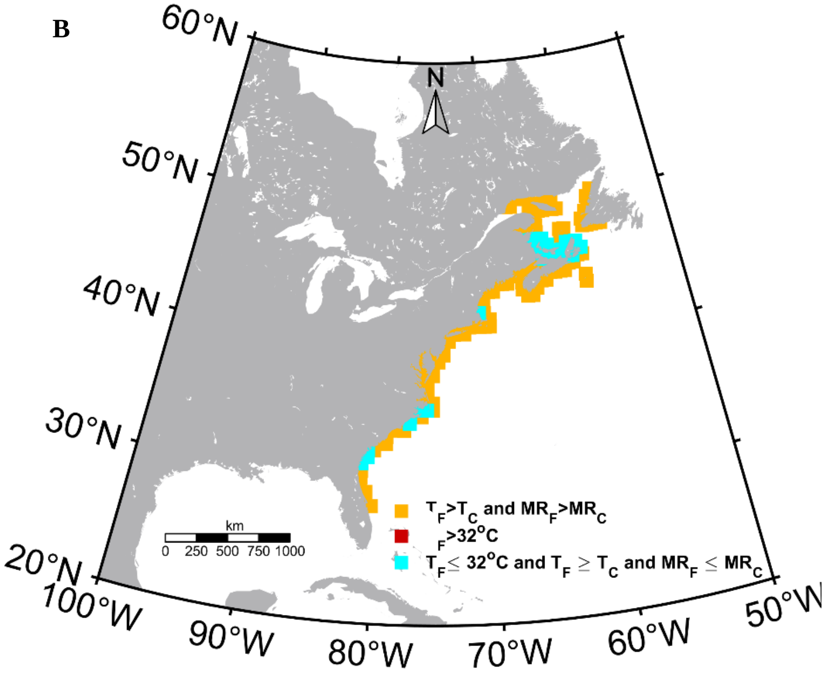

Fig. S6. Atlantic killifish populations pooled under the conditions implemented in the physiology integrated bio-climate model (PIBCM). A-C, Atlantic killifish populations pooled under PIBCM conditions in the 2050s based on data from Representative Concentration Pathways (RCPs) 2.6,4.5 and 8.5, respectively. D-F, Atlantic killifish populations pooled under PIBCM conditions in the 2080s under the same respective RCPs. In the figure legend, T is SST, F is future, C is contemporary, MR is metabolic rate range

C

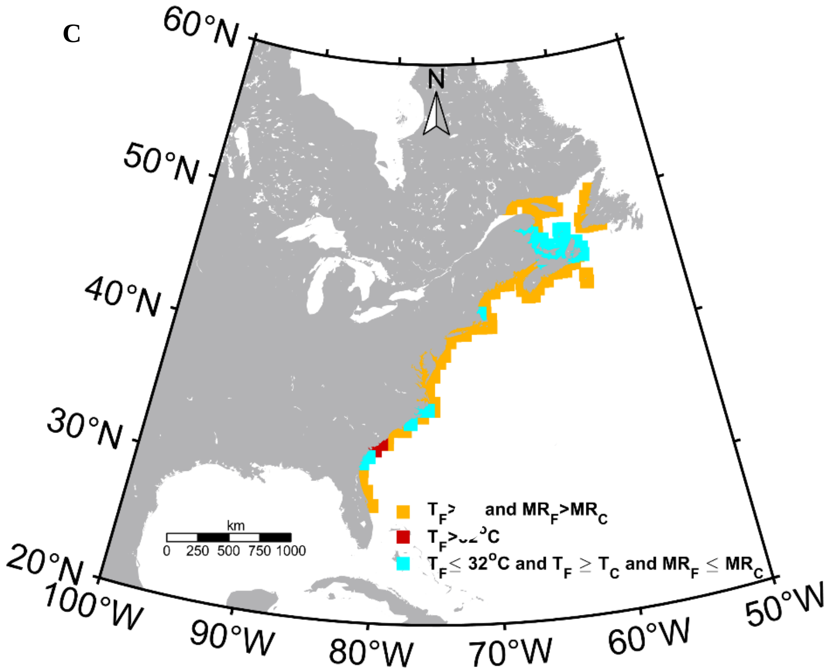

Fig. S6. Atlantic killifish populations pooled under the conditions implemented in the physiology integrated bio-climate model (PIBCM). A-C, Atlantic killifish populations pooled under PIBCM conditions in the 2050s based on data from Representative Concentration Pathways (RCPs) 2.6,4.5 and 8.5, respectively. D-F, Atlantic killifish populations pooled under PIBCM conditions in the 2080s under the same respective RCPs. In the figure legend, T is SST, F is future, C is contemporary, MR is metabolic rate range

D

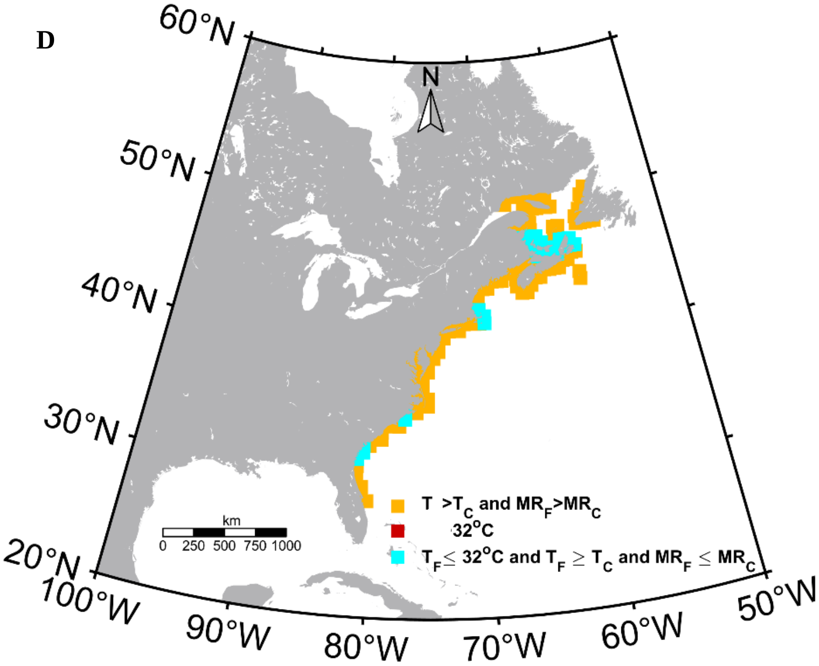

Fig. S6. Atlantic killifish populations pooled under the conditions implemented in the physiology integrated bio-climate model (PIBCM). A-C, Atlantic killifish populations pooled under PIBCM conditions in the 2050s based on data from Representative Concentration Pathways (RCPs) 2.6,4.5 and 8.5, respectively. D-F, Atlantic killifish populations pooled under PIBCM conditions in the 2080s under the same respective RCPs. In the figure legend, T is SST, F is future, C is contemporary, MR is metabolic rate range

E

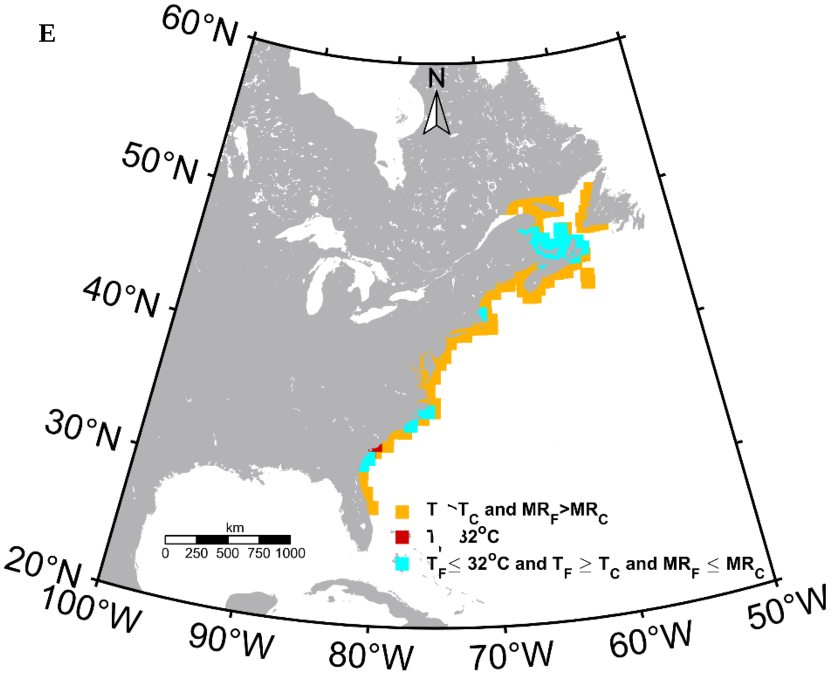

Fig. S6. Atlantic killifish populations pooled under the conditions implemented in the physiology integrated bio-climate model (PIBCM). A-C, Atlantic killifish populations pooled under PIBCM conditions in the 2050s based on data from Representative Concentration Pathways (RCPs) 2.6,4.5 and 8.5, respectively. D-F, Atlantic killifish populations pooled under PIBCM conditions in the 2080s under the same respective RCPs. In the figure legend, T is SST, F is future, C is contemporary, MR is metabolic rate range

F

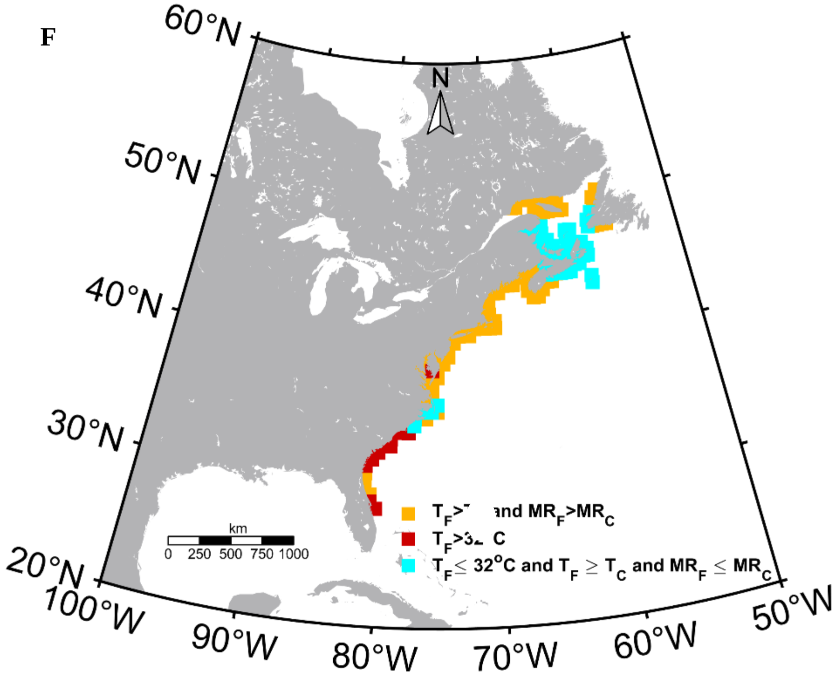

Fig. S6. Atlantic killifish populations pooled under the conditions implemented in the physiology integrated bio-climate model (PIBCM). A-C, Atlantic killifish populations pooled under PIBCM conditions in the 2050s based on data from Representative Concentration Pathways (RCPs) 2.6,4.5 and 8.5, respectively. D-F, Atlantic killifish populations pooled under PIBCM conditions in the 2080s under the same respective RCPs. In the figure legend, T is SST, F is future, C is contemporary, MR is metabolic rate range

A

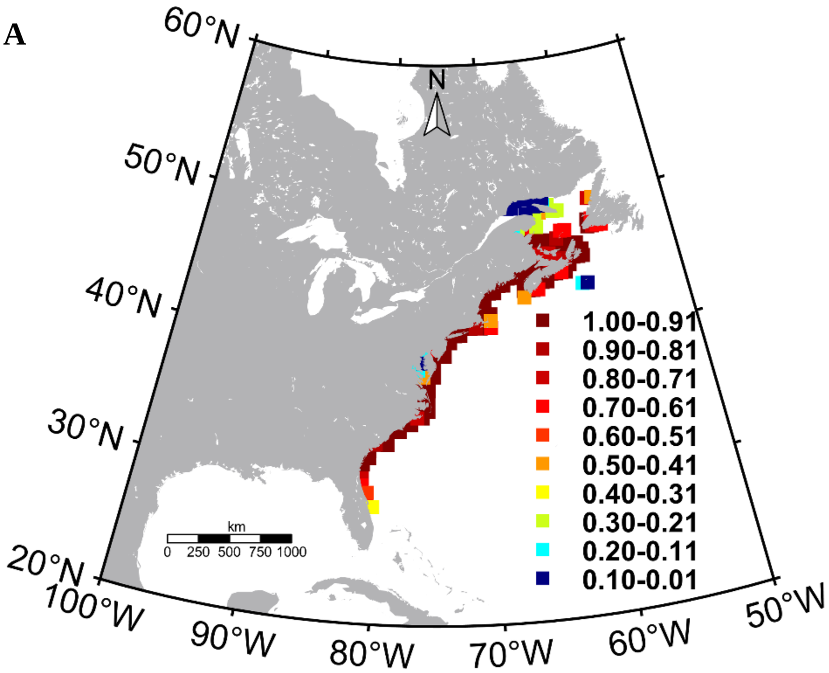

Fig. S7. Predicted Atlantic killifish probability habitat distribution in the physiology integrated bio-climate model (PIBCM) for the 2050s and 2080s based on different emission scenarios. (A) Atlantic killifish habitat distribution in the 2050s (Representative Concentration Pathway (RCP)4.5) (B) RCP 8.5. (C) Atlantic killifish habitat predictions in the 2080s (RCP2.6) and (D) RCP4.5.

B

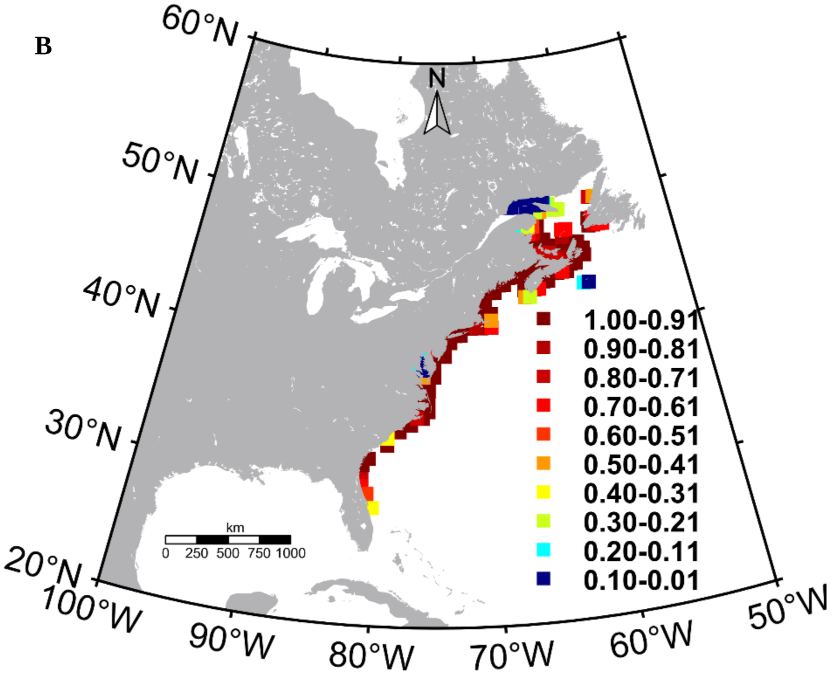

Fig. S7. Predicted Atlantic killifish probability habitat distribution in the physiology integrated bio-climate model (PIBCM) for the 2050s and 2080s based on different emission scenarios. (A) Atlantic killifish habitat distribution in the 2050s (Representative Concentration Pathway (RCP)4.5) (B) RCP 8.5. (C) Atlantic killifish habitat predictions in the 2080s (RCP2.6) and (D) RCP4.5.

C

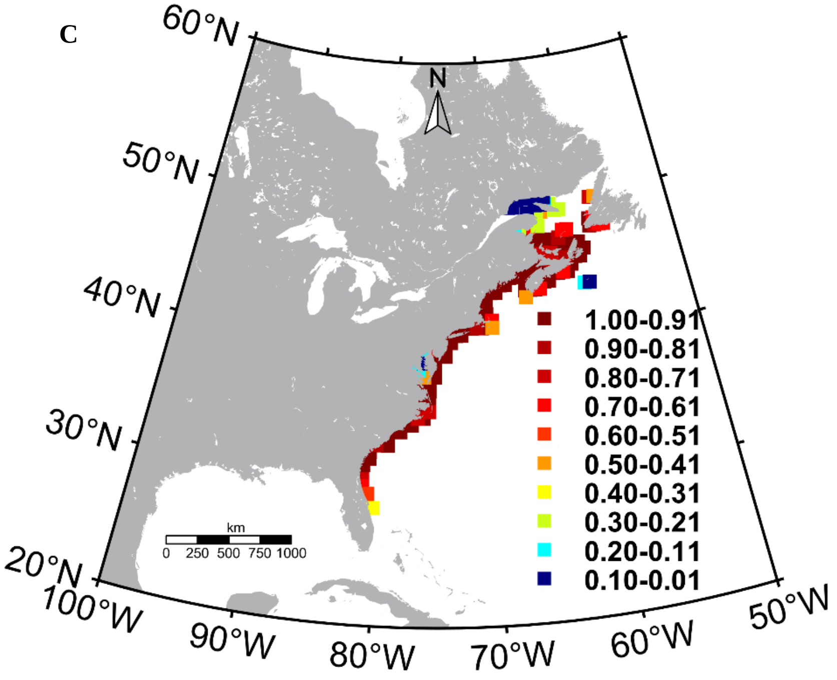

Fig. S7. Predicted Atlantic killifish probability habitat distribution in the physiology integrated bio-climate model (PIBCM) for the 2050s and 2080s based on different emission scenarios. (A) Atlantic killifish habitat distribution in the 2050s (Representative Concentration Pathway (RCP)4.5) (B) RCP 8.5. (C) Atlantic killifish habitat predictions in the 2080s (RCP2.6) and (D) RCP4.5.

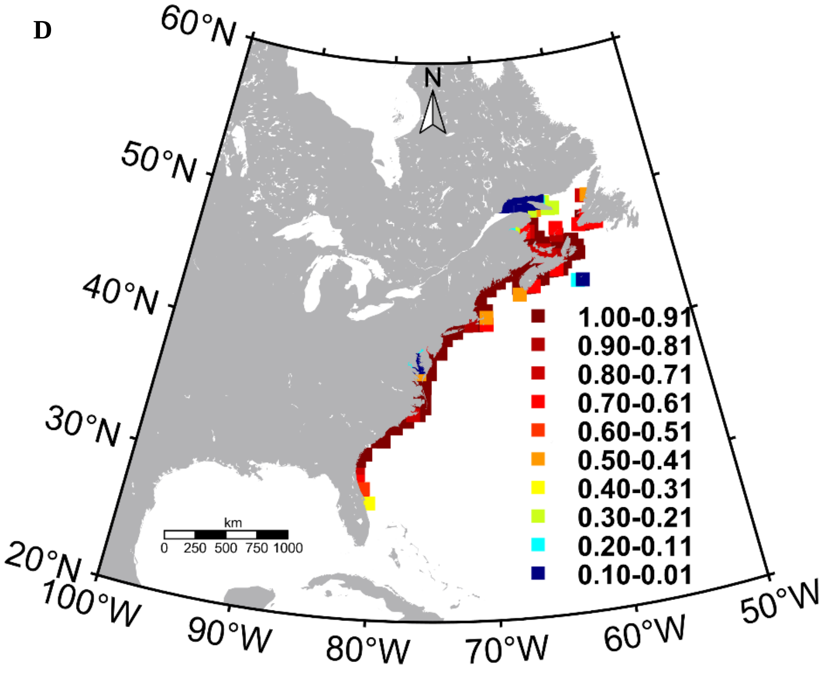

Fig. S7. Predicted Atlantic killifish probability habitat distribution in the physiology integrated bio-climate model (PIBCM) for the 2050s and 2080s based on different emission scenarios. (A) Atlantic killifish habitat distribution in the 2050s (Representative Concentration Pathway (RCP)4.5) (B) RCP 8.5. (C) Atlantic killifish habitat predictions in the 2080s (RCP2.6) and (D) RCP4.5.

A

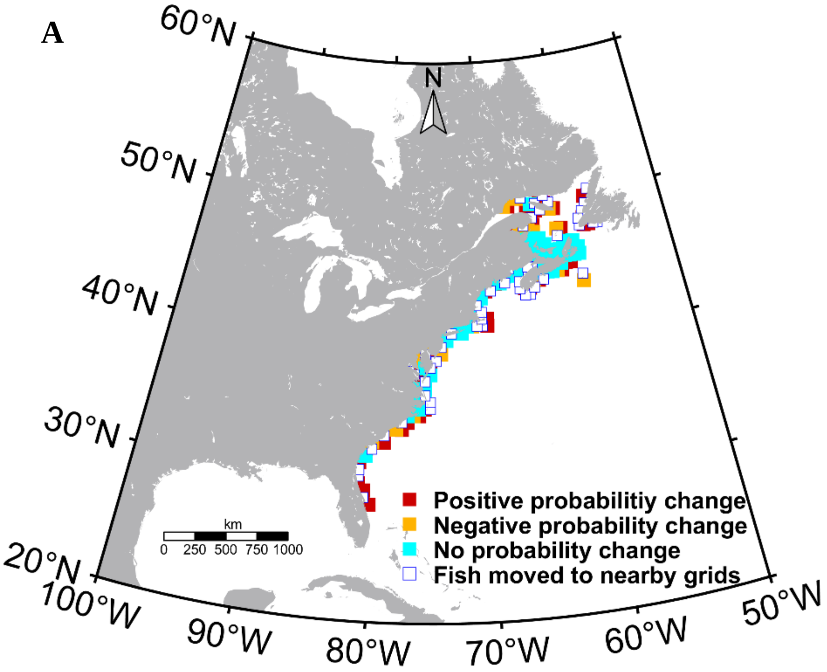

Fig. S8. Comparison between physiology integrated bio-climate model predicted Atlantic killifish habitat probabilities and the native range habitat probabilities. A-B, Comparison between Atlantic killifish native range habitat probabilities with habitat predictions for the 2050s predicted using (A) Representative Concentration Pathway (RCP) 4.5 and (B) RCP 8.5 and C-D, for the 2080s (C) RCP2.6 and (D) RCP4.5

B

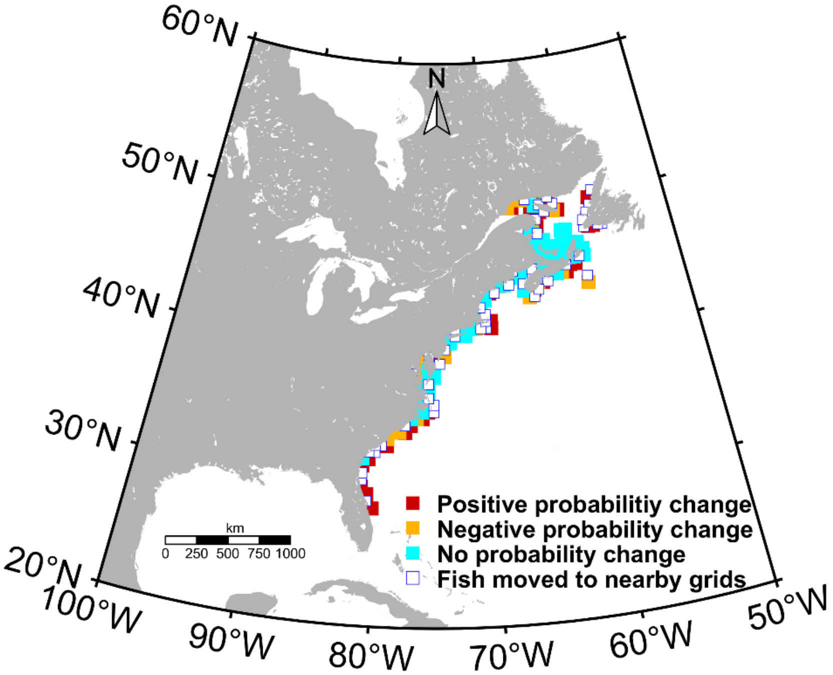

Fig. S8. Comparison between physiology integrated bio-climate model predicted Atlantic killifish habitat probabilities and the native range habitat probabilities. A-B, Comparison between Atlantic killifish native range habitat probabilities with habitat predictions for the 2050s predicted using (A) Representative Concentration Pathway (RCP) 4.5 and (B) RCP 8.5 and C-D, for the 2080s (C) RCP2.6 and (D) RCP4.5

C

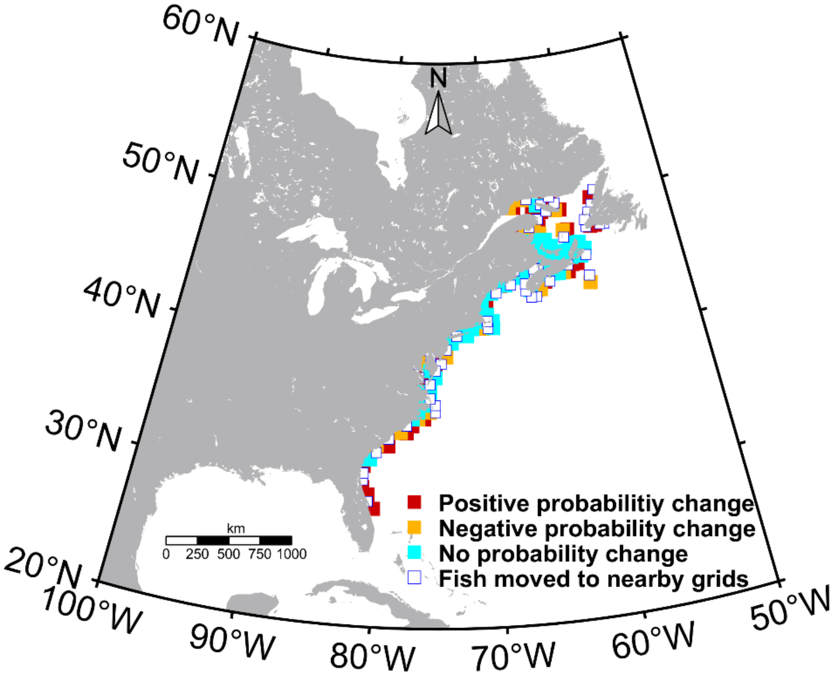

Fig. S8. Comparison between physiology integrated bio-climate model predicted Atlantic killifish habitat probabilities and the native range habitat probabilities. A-B, Comparison between Atlantic killifish native range habitat probabilities with habitat predictions for the 2050s predicted using (A) Representative Concentration Pathway (RCP) 4.5 and (B) RCP 8.5 and C-D, for the 2080s (C) RCP2.6 and (D) RCP4.5

D

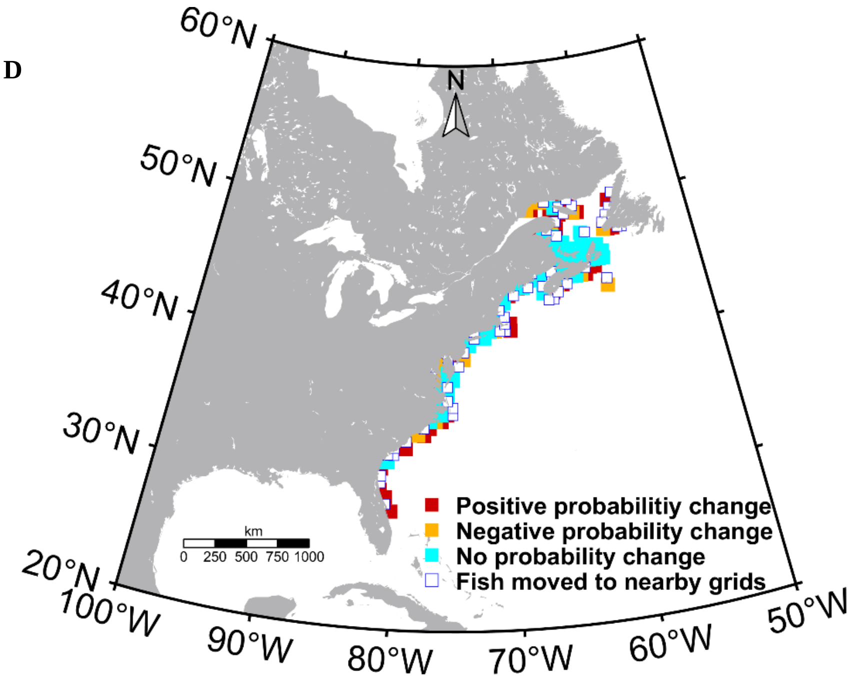

Fig. S8. Comparison between physiology integrated bio-climate model predicted Atlantic killifish habitat probabilities and the native range habitat probabilities. A-B, Comparison between Atlantic killifish native range habitat probabilities with habitat predictions for the 2050s predicted using (A) Representative Concentration Pathway (RCP) 4.5 and (B) RCP 8.5 and C-D, for the 2080s (C) RCP2.6 and (D) RCP4.5

A

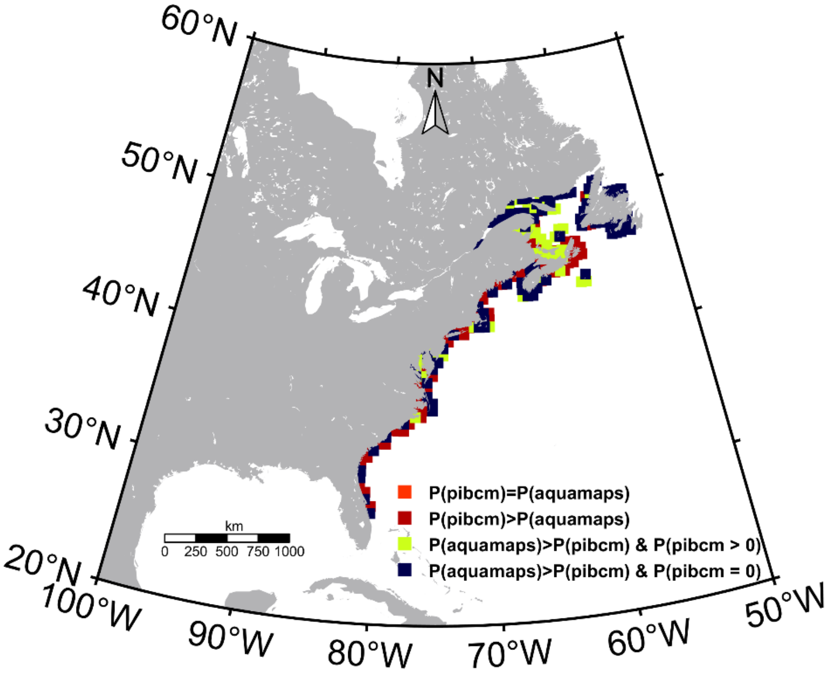

Fig. S9. Comparison between physiology integrated bio-climate model and AquaMaps predicted Atlantic killifish habitat probabilities. A-B, Comparison between two model predictions for the 2050s based on (A) Representative Concentration Pathway (RCP) 4.5 and (B) RCP 8.5. C-D, for the 2080s based on (C) RCP2.6 and (D) RCP4.5.

B

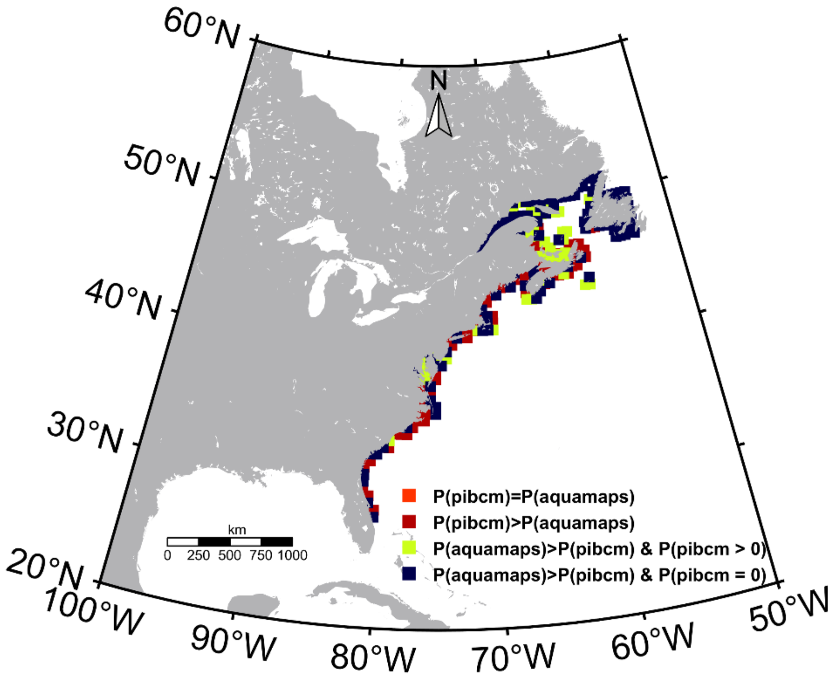

Fig. S9. Comparison between physiology integrated bio-climate model and AquaMaps predicted Atlantic killifish habitat probabilities. A-B, Comparison between two model predictions for the 2050s based on (A) Representative Concentration Pathway (RCP) 4.5 and (B) RCP 8.5. C-D, for the 2080s based on (C) RCP2.6 and (D) RCP4.5.

C

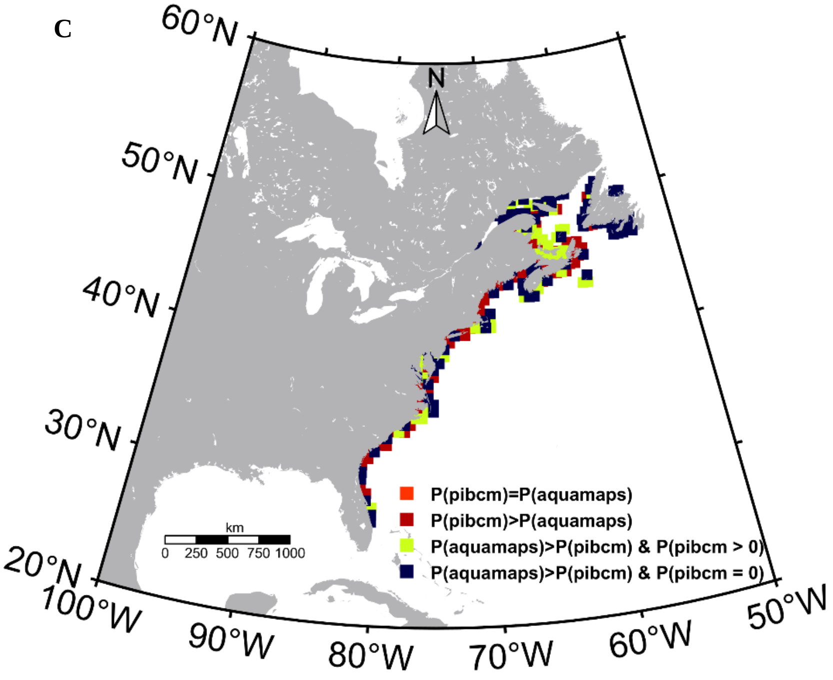

Fig. S9. Comparison between physiology integrated bio-climate model and AquaMaps predicted Atlantic killifish habitat probabilities. A-B, Comparison between two model predictions for the 2050s based on (A) Representative Concentration Pathway (RCP) 4.5 and (B) RCP 8.5. C-D, for the 2080s based on (C) RCP2.6 and (D) RCP4.5.

D

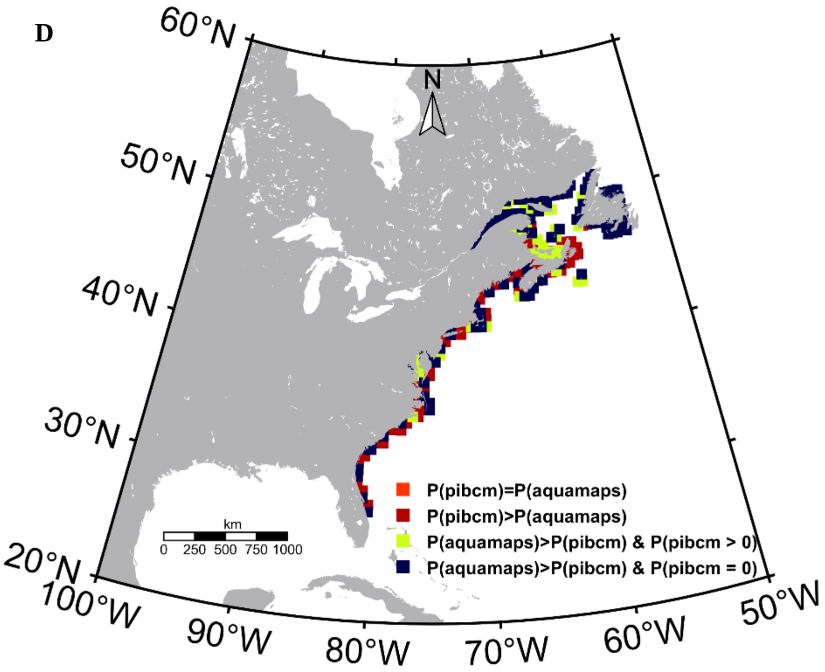

Fig. S9. Comparison between physiology integrated bio-climate model and AquaMaps predicted Atlantic killifish habitat probabilities. A-B, Comparison between two model predictions for the 2050s based on (A) Representative Concentration Pathway (RCP) 4.5 and (B) RCP 8.5. C-D, for the 2080s based on (C) RCP2.6 and (D) RCP4.5.

Table S1. List of data sources and their respective resolutions used in this study to develop the physiology integrated bio-climate model.

Data type	Resolution
AquaMap habitat probability data	0.5º×0.5º
Copernicus SST data	0.05º×0.05º
ETOPO V2- Bathymetry data	0.033º×0.033º
CMIP5 data - MPI-ESM-MR	0.8º×1.4º
CMIP5 data -GFDL-ESM2G	0.9º×1º
CMIP5 data -CCCma-CanESM2	0.9º×1.4º

Table S2. The criteria used to round the grid-specific maximum and minimum habitat sea surface temperature (SST) to Healy and Schulte,2012 assay temperatures to calculate Atlantic killifish grid-specific E values.

Condition	Rounded SST value ℃
SST $\leq$ 5	5
5 $<$ SST $\leq$ 10	10
10 $<$ SST $\leq$ 15	15
15 $<$ SST $\leq$ 20	20
20 $<$ SST $\leq$ 25	25
25 $<$ SST $\leq$ 32	30

Table S3. Calculated thermal envelope-specific E values for Atlantic killifish subpopulations in each grid based on the given maximum and minimum habitat temperatures.

Subpopulation	Temperature range	E value	*p*	r^2
Northern	5 - 10	1.01	<0.001	0.55
	5 - 15	0.85	<0.001	0.72
	5 - 20	0.67	<0.001	0.74
	5 - 25	0.7	<0.001	0.8
	5 - 30	0.67	<0.001	0.84
	10 - 15	0.68	<0.001	0.38
	10 - 20	0.49	<0.001	0.48
	10 - 25	0.61	<0.001	0.65
	10 - 30	0.6	<0.001	0.76
Southern				
	5 - 25	0.83	<0.001	0.76
	5 - 30	0.81	<0.001	0.83
	10 - 30	0.68	<0.001	0.78
	15 - 30	0.69	<0.001	0.6
	20 - 30	0.88	<0.001	0.54
	25 - 30	0.8	0.084	0.27
